# The Concept of Symmetry and the Theory of Perception

**DOI:** 10.3389/fncom.2021.681162

**Published:** 2021-08-23

**Authors:** Zygmunt Pizlo, J. Acacio de Barros

**Affiliations:** ^1^Department of Cognitive Sciences, University of California, Irvine, Irvine, CA, United States; ^2^School of Humanities and Liberal Studies, San Francisco State University, San Francisco, CA, United States

**Keywords:** perceptual constancy, invariance and symmetry, Noether's theorem, least-action principle, conservation laws, human perception

## Abstract

Perceptual constancy refers to the fact that the perceived geometrical and physical characteristics of objects remain constant despite transformations of the objects such as rigid motion. Perceptual constancy is essential in everything we do, like recognition of familiar objects and scenes, planning and executing visual navigation, visuomotor coordination, and many more. Perceptual constancy would not exist without the geometrical and physical permanence of objects: their shape, size, and weight. Formally, perceptual constancy and permanence of objects are invariants, also known in mathematics and physics as symmetries. Symmetries of the Laws of Physics received a central status due to mathematical theorems of Emmy Noether formulated and proved over 100 years ago. These theorems connected symmetries of the physical laws to conservation laws through the least-action principle. We show how Noether's theorem is applied to mirror-symmetrical objects and establishes mental shape representation (perceptual conservation) through the application of a simplicity (least-action) principle. This way, the formalism of Noether's theorem provides a computational explanation of the relation between the physical world and its mental representation.

We begin with a perceptual illustration that will motivate the rest of this paper. Consider the 2D image of a transparent cube, shown in Figure 1A. When one looks at this Figure, one sees a 3D object, a transparent cube. A cube is a highly symmetrical object: it is characterized by reflectional, rotational, and central symmetries. The symmetry of an object is defined as the invariance of the object under transformations. For example, rotating a cube by a multiple of 90 degrees around an axis that connects the centers of two parallel faces results in the same cube in the same 3D position and orientation. Similarly, reflecting the cube about a plane that splits the cube into two identical halves, results in the same cube. It is now known that the symmetry of 3D objects is of fundamental importance in seeing 3D objects as 3D (Pizlo et al., 2014). Next, look at Figure 1B, which is a 2D image of a 3D polygonal line. This 3D polygonal line connects the eight vertices of a cube shown in Figure 1A in random order. The cube in Figure 1A is symmetrical, but the 3D polygonal line in Figure 1B is not symmetrical. It turns out that, when only one 2D image is available and a 3D object has no trace of symmetry, this object is not perceived as 3D (see the polygonal line stimuli in experiments by Edelman and Bülthoff, 1992; Pizlo and Stevenson, 1999; Chan et al., 2006; Li and Pizlo, 2011. See also the bent wire stimuli in Rock et al., 1981, 1989; Rock and DiVita, 1987). However, when the 3D object is symmetrical, human observers always perceive it as 3D and they perceive it veridically (Pizlo and Stevenson, 1999; Chan et al., 2006; Li and Pizlo, 2011; Jayadevan et al., 2018). By “veridically” we mean that observers perceive the 3D shape the way it is out there. In other words, the perceived shape matches, or nearly matches the physical shape (Pizlo et al., 2014). One may ask, how can we ever know that an observer perceives a cube veridically? After all, perceptual representations are private mental events. Symmetry comes to the rescue here. If the observer says that the object is formed by 3 pairs of parallel faces, and the object has 6 planes of mirror symmetry, we know that the observer perceives a cube. If there are only 3 planes of symmetry, and these planes are parallel to the faces of the object, the observer is looking at a shoebox. With 5 planes of mirror symmetry, it is a pizza box (or a square rod, like a leg of an ordinary chair). By describing the shape of a physical object in terms of its symmetries, the private mental representation of the observer has been made public, in the sense that we all know what the observer's percept is. This is not surprising in our everyday life: after all, we rarely wonder about whether what we see is the same as what others see. Symmetry and its invariance properties are essential in human vision, as well as in other cognitive functions. Without symmetry, vision, as we know it, would not exist (Pizlo, 2019). By the time the reader finishes reading this paper, they should become familiar with the multiple facets of symmetry and invariance. It is no coincidence that symmetry permeates nature at all of its levels.

**Figure 1:**
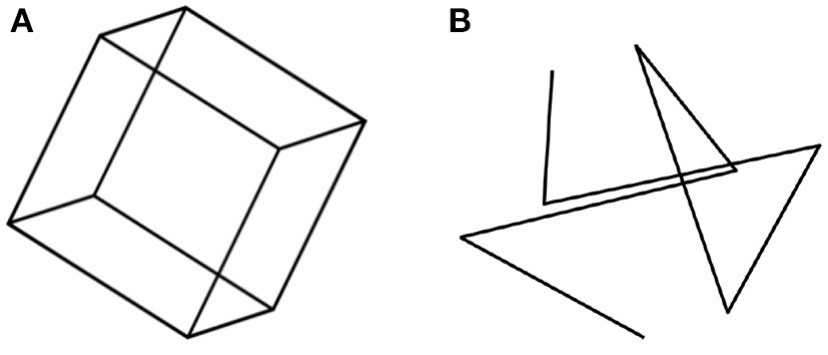
**(A)** shows a transparent cube that is perceived as a 3D cube because of the cube's multiple symmetries. **(B)** shows a polygonal line connecting the vertices of a cube. **(B)** is not likely to be perceived as a 3D polygonal line because of the absence of 3D symmetries (from Pizlo, 2008, MIT Press).

## 1. Invariance in Natural Science

Invariance in natural science is a fundamental concept. By invariance in science, we mean that a given law or property stays unchanged (is symmetric) under certain transformations. For example, Newton's second law of motion^1^ (**F** = *m***a**), represented by a mathematical relation, does not change when we apply this law in different places or different times, i.e., this law is invariant (symmetric) under spatial displacement and temporal translation.

Let us consider an example. Let us apply a constant force F along the *x*-axis to an object at rest and with mass *m* and observe how far the object moves after a time interval δ*t* relative to the starting point, whose coordinate is marked on the floor as (0, 0). For simplicity, we assume that this object is restricted to move on the horizontal surface (*x, y*) and that the only (horizontal) force acting on it is **F**. According to Newton's Second Law, the object's acceleration **a** is given by **a** = **F**/*m*, and for a constant force the distance traveled can be computed as $d=a_{x}t^{2}/2$, where $\text{a}=a_{x}\hat{\text{x}}=\left(\frac{F_{x}}{m}\right)\hat{\text{x}}$ and $\hat{x}$ is the unit vector in the direction *x*. The final position of the object after time *t* is (*x, y*) = (*d*, 0). Now we repeat the experiment, but we shift the object by two meters along the *x-*axis, with the new starting point being at (2, 0). This shift means that we translated the experiment by two meters. If we apply the same force, after time *t*, our object moves by the same interval *d*, so its final position is (*x*′, *y*′) = (*d* + 2, 0). Instead of moving the object two meters along the *x-*axis, we could move the observer and their reference frame, such that in the new observer's vantage point, the object would not be initially at coordinates (0, 0) but instead at coordinates (2, 0). Regardless of what we do, the result *x*′ of a translated experiment is the same as the translated result of the first experiment, i.e., *x*′ = *x* + 2. Notice that the total distance traveled, defined as the absolute value of the difference between the final and starting points, is the same for both situations, namely *d*. This is an example of what is called in physics symmetry of the Natural Law, in this case, a translational symmetry. So, Newton's Second Law is invariant under translation.

**Figure 2:**
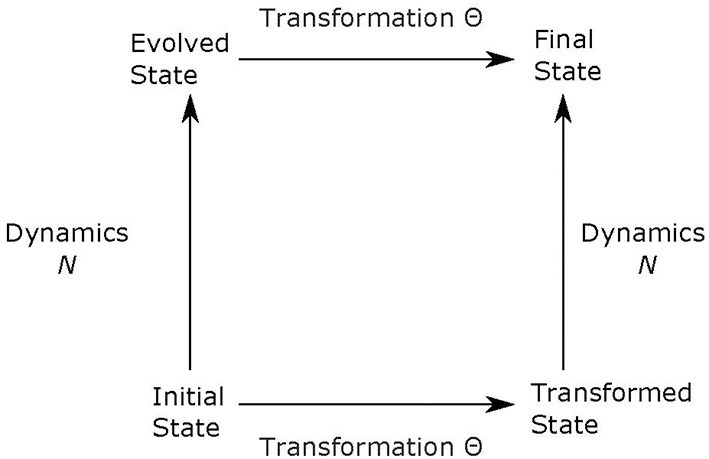
The transformation Θ is a symmetry of the dynamical evolution *N* if, for any initial state u, the final state when first evolved and then transformed is the same as the one obtained by first transforming and then evolving, i.e., *NΘu* = Θ*Nu*.

Following Rosen's (2008, pp. 264–266) notation, if *N* is our natural law (in our case, Newton's second law), which determines how the initial state *u* evolves into the final state, and Θ represents a transformation (in our example, translation), then *NΘ*(*u*) = Θ*N*(*u*). What this means, applied to our example, is that if we move the coordinate system by two meters and then use Newton's Law, we get the same outcome as if we just apply Newton's Law first and then move the coordinate system by two meters. Accordingly, physicists associate symmetries to invariances of the laws of nature. It has been commonly agreed that there would be no natural laws and no science without symmetry (Wigner, 1967; Rosen, 2008). Before Einstein's special relativity theory, it was assumed that the symmetry (invariance) of a physical law should be derived from experimental results or the law itself. Einstein reversed this logic when he formulated both relativity theories. He assumed that symmetry (invariance) comes first, and the new laws should be formulated so that they satisfy the symmetries. This new approach to how theories are formulated has been universally accepted by physicists. This general statement is often illustrated in a diagram like that in Figure 2.

Consider another example illustrating the symmetry of natural laws (Feynman, 1965). When the Moon revolves around the Earth, the gravitational force between the Moon and the Earth causes the Moon to fall toward Earth—by saying that the Moon falls toward Earth, we mean that the (almost) circular trajectory of the Moon falls below the straight line that it would have been in if there was no force acting on the Moon. It is easy to calculate that the Moon falls toward the Earth about 1.4 × 10^−3^m in 1 s. The Moon is 60 times as far away from the Earth's center as we are, so if the inverse square law is right, the object at the Earth's surface should fall in one second by 1.4 × 10^−3^m · 60^2^, which is 4.9 m. It has been known already from Galileo's measurements that things fall in 1 s by 4.9 m on the Earth's surface. So, the fall's magnitude is not invariant under translation from the Moon's orbit to the Earth's surface, but the magnitude of the fall of an object on the Earth's surface (in Rosen's, 2008 terminology, it is the result of a transformed experiment), can be computed by transforming the magnitude of the Moon's fall through the inverse square law (in Rosen's terminology, it is transformed result of an experiment): *NΘ*(*u*) = Θ*N*(*u*). For additional discussion of invariance in physics see Appendix A in Supplementary Material.

### 1.1. Invariance in the Natural Environment

Physical theories and their laws are somewhat abstract, as they refer to abstract objects, such as point particles, ideal forces, constant masses. But the physical world consists of concrete objects, such as planets, rocks, buildings, cars, chairs, fluids, windows, trees, animals. The theories need to be related to the actual objects through the construction of physical models that describe the system of interest. For instance, Newton's Principia showed that the movement of, say, a planet could be described, in part, by the motion of its center of mass. The planet's center of mass is a mathematical point that accelerates at the rate **a** = **F**/*m*, where *m* is the mass of the planet, and F *is the sum* of all forces acting on it. So, instead of dealing with the complications of a large number of particles, as it is the case of a planet, we can model its motion by using the center of mass.

In this sense, physics is not about objects, but abstract characteristics of objects, such as its center of mass, total mass, and so on, although the laws of physics can be applied to objects. For example, Archimedes's law of the lever, Galileo's law of falling bodies, Kepler's laws of planetary motion, or Bernoulli's law for fluids and gases, they all can be, and are, used to describe how physical objects can be manipulated or observed in experiments and how they can be used in engineering projects, such as building houses, bridges, or cars. Objects reside in the physical world, and so, invariance of physical laws applies to them, as well, although invariance may not be guaranteed. As long as a system is isolated, its energy is conserved, but truly isolated systems do not exist (they are approximations). Similarly, as long as a chair is not broken or destroyed, it is invariant under certain transformations, as for rigid bodies only six degrees of freedom are necessary for their description (the center of mass and Euler angles to describe the orientation of the rigid body), although they are composed of an enormously large number of particles (atoms). Similarly, a rectangular block of ice is invariant under rigid motion, but once it melts, it loses some of its invariant properties, like shape and volume.

When we talk about the invariance of objects, we refer to their permanent characteristics under certain assumptions of a model: their shapes, sizes, masses, surface reflectances, and viscosities. In fact, without the permanence of objects, there would be no science of physics because the laws of physics were inferred by scientists through the manipulation of permanent objects. Systematic experiments would be impossible without prisms that refracted and dispersed light, inclined planes and marbles that rolled on these planes, cannonballs that could be dropped from the leaning tower of Pisa, paints that could be mixed to produce a range of colors, resistors that are used in electrical circuits to test Kirchhoff's current and voltage laws, and so on. The permanence of objects is implied by the laws of physics, which themselves are governed by the invariance principle. At the same time, the permanence of objects implies their invariance under some transformations. From now on, whenever we refer to the invariance of objects, we will also talk about the corresponding transformations of these objects. It is impossible, or at least unscientific, to speak about invariance without specifying the relevant group of transformations.

We emphasize that invariance makes it possible for physicists to recognize patterns and laws in nature because they allow for certain phenomena to be predictable. This predictability, of course, is not a property of the physical laws, but the observed systems. For example, one could imagine an astronomer on a planet such as Star War's Tatooine, which orbits a binary system. As Alekseev (1969) showed, some simple binary systems are so chaotic that their observed symbolic dynamics is isomorphic to a coin-toss. So, in Tatooine, astronomical predictions, depending on the planetary dynamics, maybe hopeless. It is not that the laws of physics do not apply: they do. It is that in such systems, the dynamics are so complicated as to be, in practice, unpredictable. An astronomer in a binary system may have no hope of coming up with Kepler's Laws. To them, the laws of motion for the heavenly bodies would appear to be random. In other words, symmetries and invariance are essential, but predictability is also crucial. When one looks at a house today and then again tomorrow, there is an invariance that allows for the predictability of the house's shape, and the recognition that it is the same house.

Not all objects are invariant and rigid. For example, animal bodies are not rigid, but they are both important and common in our environment. Animal bodies are approximately piecewise rigid. What this observation means is that with animal bodies one has to use a group of transformations that is more general than a group of 3D rigid motion. That is the only generalization that is needed. Animal bodies are characterized by invariant properties and they are also characterized by redundancies. For example, human bodies are topologically mirror-symmetrical regardless of articulation of limbs. Limbs can form a wide range of articulations, but the possible articulations are limited by biomechanical constraints. A natural walking of a person can be viewed as a glide reflection in a 3D space plus time (the pose when the right foot is forward is a 3D mirror reflection of the pose when the left foot is forward). All this means is that the case of animal bodies can also be treated by the formalism based on groups and invariants. There is an additional generalization. The proportions and shapes of body parts change during the childhood and adolescence. These changes could also be incorporated in our theory: body parts do change but the changes are not completely arbitrary. A discussion of shapes of plants and animals during the growth process has been reviewed in a classical book by Thompson (1942), titled “On Growth and Form.”

### 1.2. Invariance and Group Theory

Consider a transformation called rigid motion, which is common in our environment, and intuitively obvious. The technical meaning of “rigid motion” is the same as its colloquial meaning. Rigid motion in a 3D space includes 3D rotations and 3D translations. Rigid motions form a group of transformations, where by “group” we mean that a set *T* of transformations satisfies four properties (axioms):

(i) an identity transformation *I* that does nothing is in the set *T*;
(ii) each transformation *t* ∈ *T* can be undone by a transformation *t*^−1^ ∈ *T* called the inverse of *t*;
(iii) a composition of two transformations *t*_1_ and *t*_2_ is a transformation that is in the set *T* (this is a closure axiom), i.e., if *t*_1_, *t*_2_ ∈ *T*, then *t*_1_*t*_2_ ∈ *T*; and
(iv) grouping transformations does not change the result, i.e., *t*_1_(*t*_2_*t*_3_) = (*t*_1_*t*_2_)*t*_3_ (this property is called associativity).

As a simple example, we can show that one-dimensional translations form a group under the operation of addition. For axiom i, we observe that for any number *n*, the number 0 gives the identity transformation, as *n*+0 = *n*. Also, for any number *n*, −*n* is the inverse transformation, as the transformation *m* + *n* = *k* if followed by the transformation *k* − *n* gives back *m* (axiom ii). Additionally, for any numbers *m* and *n*, adding *m* and then adding *n* is the same as adding (*m* + *n*), which gives iii. Finally, for any three numbers it is true that *m* + (*n* + *k*) = (*m* + *n*) + *k*, which corresponds to axiom iv.

When a set of transformations is a group, there is an invariant characterizing this group (Weyl, 1966). The converse is true, as well. It is easy to realize that pairwise distances are invariant under rigid motion. Angles are invariants of rigid motion, too, but they are also invariant under a larger group, called similarity, a group of rigid motions and uniform size scaling.

## 2. Invariance in Perception

Symmetry (invariance) also characterizes the Laws of Cognitive Science, but note that the cognitive community does not use the formalism of symmetry, nor has it explored its implications. Now take an example from visual perception. When we are in front of our house, and we look at it today and tomorrow (note that we examine here the Laws of Perception in the presence of translation along the time-axis), our 3D percept of the house is the same on both days. So, our percept is symmetric (invariant) in the presence of translation along the time-axis.

Already Cassirer (1944) pointed out that empiricists, such as von Helmholtz (1924/1886), ignored or downplayed the operation of symmetry in the Laws of Perception. If our perceptions, including the mechanisms that produce perceptions, were a direct result of accumulating perceptual experience, our percepts today and tomorrow could not be guaranteed to be identical. Similarly, the percept of our guests, when they look at our houses when standing in front of them would most likely be different from ours because we and they, like any individuals, have had different life experiences, including the fact that we looked at our houses more times than they probably had. Clearly, the *nativistic* tradition in perception is more favorable to the concept of invariance in the perceptual laws.

According to nativists, all perceptual mechanisms are innate. The mechanisms are hardwired, rather than learned, and they are not modified during the person's lifetime. Obviously, some parameters are likely to be adjusted during personal development, like interocular distance. But vision algorithms stay the same. Similarly, the phenomenon of perceptual constancy is defined precisely as the ability to see the object's characteristics such as shape, size, and color as the same from one presentation to another (Walsh and Kulikowski, 1998). Perceptual constancy may fail under extreme viewing conditions. These failures cannot be surprising and they do not invalidate perceptual constancy. Three-dimensional vision is an inference based on incomplete 2D retinal data through the application of a priori constraints (Pizlo, 2001). When the stimulus dramatically violates the constraints, the percept will not be constant. In other words, the theory makes predictions as to when perception can be “tricked” in the form of illusions. Look at the Ames's chair demo: http://shapebook.psych.purdue.edu/1.3/. This image is perceived by a human observer as an image of a chair, when in fact this image was produced by a set of 6 unconnected parts. You can see the 3D stimulus consisting of unconnected parts by rotating it with your mouse. Note that even though you now know that this is not a chair, you cannot help, but perceive a chair from the original image. It is tempting to explain this illusion by referring to familiarity with chairs. But in our theory familiarity is not needed. Your percept of an object, we all call chair, is the only 3D symmetrical interpretation of the asymmetrical 2D image. Even if you know that the object in front of you is not symmetrical, you perceive it as symmetrical (see Pizlo et al., 2014 for more details and more examples testing and supporting this theory). What is important for our argument is that perceptual constancies are satisfied most of the time in our daily lives. As Cassirer (1944) pointed out, it was natural for nativists such as Plato, Kant, Hering, Poincare, and Weyl to consider the operation of symmetry and invariance in the laws of perception in particular, and in the laws of cognition in general, simply because nativists always emphasized the importance of *a priori* abstract mathematical concepts, called “universals.” Invariance is such a universal. Perhaps, it may even be the most important universal. The main goal of this paper is the re-evaluation of the role of symmetry and invariance in Psychology, and their relevance to the status of our field among the Natural Sciences.

We just pointed out that the laws of perception are likely to be symmetric (invariant) under the transformation from 1 day to another or from one observer to another. Without this symmetry, the science of perception, like the science of physics, would not exist. Perception as a cognitive phenomenon would probably not exist, either, at least not the way we understand it. If your perception of a cube kept changing from moment to moment, such as seeing a cube today but a sphere tomorrow, and if different observers saw different objects when looking at the same object, then it would not make sense to talk about perception. Perception would serve no useful function.

**Figure 3:**
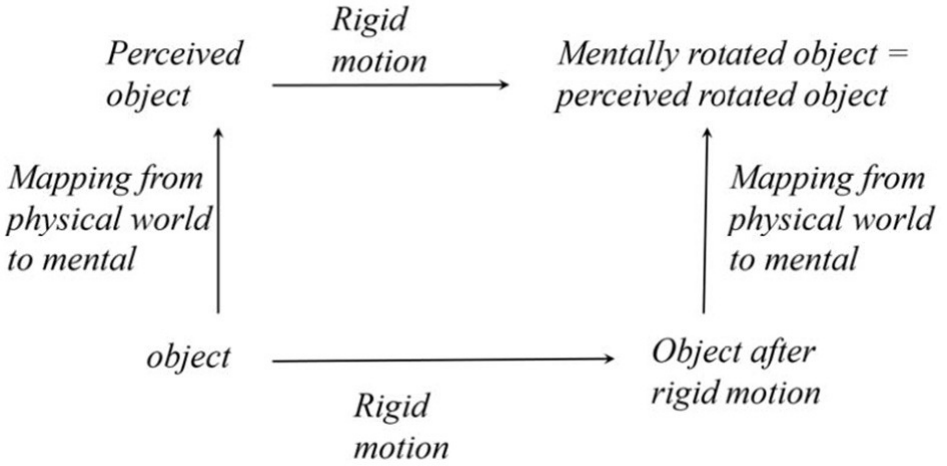
A 3D object is invariant under rigid motions, and the perception of this object is invariant under the same group of rigid motion. This invariance implies that the observer perceives the permanent characteristics of this object. We used a label “mental rotation” instead of “mental rigid motion” because the former has already been used in the cognitive literature.

Next, we will examine the invariance of perception more specifically. Namely, we will consider the perception of objects when they undergo spatial transformations. So, instead of comparing our perception of our house today and tomorrow, which is the kind of temporal invariance we discussed earlier in this paper, we will compare our perception of our car when we look at it from two different viewing directions, which happens when the car undergoes rigid motion. If perception does not change despite the transformation of an object, we call this perceptual invariance “perceptual constancy.” This case is essential because it is defined as perceiving permanent characteristics of objects when the objects are subjected to transformations representing a group. Transformation groups have invariants. These invariants define the permanent characteristics of objects. If we can veridically see the permanent characteristics of objects, our perception is invariant (constant) the same way objects are invariant. This relation is illustrated in Figure 3. Note the similarity of the diagrams in Figures 2, 3.

But, if an object's invariance is established by applying transformations to the object, perceptual invariance can only be verified by applying transformations to the perceived object as well. Shepard and his colleagues' description of mental rotation is a perfect example (Shepard and Metzler, 1971; Shepard and Cooper, 1982). That we can imagine looking at a given object from a different viewing direction means we mentally rotate the object. Similarly, we can see a given object as the same, although the object has been physically rotated, and we see that the object was rigidly rotated. This is a commonly accepted definition of perceptual constancy. At the same time, this definition specifies how perceptual constancy should be handled in experiments. Without clarifying what group of transformation is being evaluated, the experiment may or may not be meaningful. The next section will briefly review some classical attempts to verify size, speed, shape, lightness, and color constancy. These five constancies are essential because they are directly related to the permanent characteristics of objects in the presence of changes in the viewing conditions.

### 2.1. Prior Work on Perceptual Constancies

Conventional approaches to perceptual constancies have focused on the role of contextual cues on the perceived characteristics of objects such as size, shape, and reflectance (lightness and color). The context was represented by the viewing distance, the object's 3D orientation relative to the observer, and the intensity and spectrum of the illuminating light. The context did represent the transformations of the object, but the associated groups of physical transformations have never been explicitly evaluated. Previous studies of perceptual constancies explored only subsets of the relevant groups and rarely (if ever) examined the groups' invariants. This emphasis on the role of context, rather than invariants, had two undesirable consequences. First, instead of documenting under what conditions perceptual constancy is achieved, the previous studies focused on documenting which manipulations of the context would harm or eliminate the constancy. Second, the relevant transformations were often excluded from experiments, making it impossible to treat and study constancy as an invariant of transformations. This emphasis has been, and still is, fairly common in the case of shape constancy. In this section, we describe a few studies that illustrate our point, and we do not mean for it to be comprehensive.

#### 2.1.1. Size and Speed Constancy

Let us begin with perceptual size constancy. If the object's perceived size is the same, despite viewing the object after the object underwent 3D rigid motion relative to the observer, the observer achieved size constancy. Prior research on size constancy focused almost exclusively on the effect of the viewing distance on size constancy. Viewing distance is only one out of 6 parameters characterizing 3D rigid motion. Ignoring the other two translations, up-down and left-right, seems justified because these two translations have a small effect on the retinal size. But there is no reasonable justification for ignoring 3D rotations in size constancy experiment. Again, rotation around the line of sight is less appealing because this rotation does not change the retinal size. But the other two parameters of 3D rotation should have been included in size constancy experiments. But they were not, except for a very recent study by Maruya and Zaidi (2020). This restriction of size constancy experiments has never been justified, as if it were intuitively obvious. It was not.

If the retinal size in the observer's eye is measured conventionally by the viewing angle α, then tan(α) = *S*/*D*, where *S* is the linear extent of the object measured in the direction orthogonal to the line of sight and *D* is the viewing distance. Size constancy is fairly reliably achieved, both monocularly and binocularly, under natural viewing conditions (Holway and Boring, 1941). Specifically, the systematic error is small, but the random error (variability of responses from trial to trial) is substantial.

Speed constancy experiments used an identical experimental method as size constancy (McKee and Smallman, 1998). The only manipulation used in the past was the size of the reference frame that was assumed to be related to the viewing distance. No other parameters of 3D motion were manipulated. Manipulating the other parameters of rigid motion in the case of speed constancy is at least as interesting as in the case of size constancy. Consider an object moving with a constant speed along a straight line. Does an observer perceive the speed as constant, despite the fact that the retinal speed changes, due to perspective projection, as a function of position and time? If human observers have intuition about the Laws of Physics, we would expect that they would demonstrate speed constancy in such an experiment. To the best of our knowledge this has never been tested.

#### 2.1.2. Shape Constancy

Now, consider the perceptual shape constancy. Shape constancy has a long history that started one thousand years ago (Alhazen, 1989). This history has been reviewed by one of us in two monographs (Pizlo, 2008; Pizlo et al., 2014). Here, we will focus on the recent work in which the concept of invariance played a decisive role. This recent research started with Biederman and Gerhardstein's (1993) paper where they demonstrated that shape perception is invariant under 3D rotation.

Biederman and Gerhardstein presented the subject with a 3D shape on the first presentation, they then rotated the shape around the axis that was orthogonal to the subject's line-of-sight, and presented it again. The subject's task was to recognize whether the 3D shape was the same on both presentations. In half of the trials, the two 3D shapes presented were different. The two different shapes differed by having different parts. Thinking about qualitatively different parts, encouraged Biederman to claim that shape perception can only be explained by specifying topologically different features. Biederman was not prepared to claim that humans have perceptual access to the group of rigid motions and its invariants. But the issue as to whether perceptual constancy is real or not should have been settled about three decades ago. It was not for some obscure reason.

Why are so many contemporary students of vision still arguing whether shape constancy is real? The main reason (although not the only reason) for the existing confusion surrounding shape constancy is the fact that shape constancy has not been treated as a perceptual invariant related to a group of transformations. But the only way to make sure that you are studying shape as an invariant of rigid translation and rotation is to have the subject look at the object after it has been subjected to rigid translation and rotation, the way it is illustrated in Figure 3. Biederman's experiments, and experiments performed by one of us (Pizlo and Stevenson, 1999; Chan et al., 2006; Li and Pizlo, 2011), followed that invariance principle. No other recent experiments did. We hope that this argument based on invariance is commonsensical to the reader. More importantly, however, this argument follows the method that Physicists have been using for over a century—compare the diagrams in Figures 2, 3.

Imagine that you show a 3D object from a single 3D viewing direction and ask a subject to judge the entire shape or its local 3D features like the lengths of line segments, or angles formed by pairs of line segments or pair of surfaces, or local surface orientations. If no other viewing direction is used, the subject is presented with an anisotropy because the depth dimension is visually much less accurate and less reliable than the other two dimensions. In fact, with a monocular viewing from a single 3D direction, the visual system obtains no direct sensory data about the depth dimension (except for the accommodation depth cue that can be eliminated when a 2D image is used).

With binocular viewing, direct depth measurements are available, but they are almost always biased and unreliable. One of the central theoretical claims of the present paper, a claim that follows a 35-year-old tradition in vision science, is that the 3D shape percept is produced by combining 3D a priori constraints, such as symmetry, compactness, and planarity, with the 2D retinal image (Poggio et al., 1985; Pizlo, 2001, 2008, 2019; Pizlo et al., 2014). Sensory depth information is not necessary in such models, although depth information can be used if this information is reliable (see Li and Pizlo, 2011; Li et al., 2011; Jayadevan et al., 2018). So, if perceptual depth measurement is not used in producing a 3D shape percept, how can the accuracy of shape perception along the depth dimension be verified? The answer is that it must be verified by showing another view of the 3D object and asking the participant whether the 3D shape is the same in both views. If the same 3D shape was used in both presentations and the two shapes are perceived as identical, shape constancy has been established. This is precisely how shape constancy is defined in perception textbooks (see Walsh and Kulikowski, 1998). If the second view is not used, the experimenter runs a risk of measuring not just shape perception but a confound of (conflict between) shape and depth perception (this can also be referred to as a conflict between a priori constraints and depth cues, such as binocular disparity—see Figure 10 in Li et al., 2011). When shape perception is tested as an invariant of rigid motion (the way Biederman and Gerhardstein, 1993; Li and Pizlo, 2011 did), shape constancy is very reliable, and the shape percept is much better than depth percept (Pizlo, 2008). The fact that shape can be perceived more reliably than depth comes as no surprise to computational modelers because it is well known there are several effective constraints for shape but not for depth. The bottom line is as follows: the 3D shape is invariant under 3D rigid motion and must be tested as such. The subject must be shown a 3D object from more than one 3D viewing direction in order to verify whether the perceived shape itself is invariant under 3D rigid motion.

We conclude our treatment of shape constancy with a brief comment about constancy experiments that used unstructured, amorphous objects. Once we acknowledge that 3D shape perception is based on applying a priori constraints, such as 3D mirror-symmetry, to the 2D image(s), it follows that 3D shapes will not be perceived veridically if the constraints are ineffective. This was true with Rock's wire objects (Rock et al., 1981, 1989; Rock and DiVita, 1987) and (Edelman and Bülthoff, 1992) bent paperclips and random star shapes. It so happens that objects in our real environment never (or almost never) lead to violations of shape constancy: a priori constraints are effective because symmetries characterize objects in our environment.

At this point, the astute reader is surely asking now the next important question: what is hiding under the label “mapping from the physical world to mental” in Figure 3? The answer will come shortly from examining the nature and implications of the seminal theorem of Noether (1918) that set the stage for placing symmetry at the center of modern Physics. But before we describe what Noether accomplished, we will briefly describe experiments on two other perceptual constancies.

#### 2.1.3. Lightness Constancy

Lightness constancy refers to the ability to judge the albedo (% reflectance) of a surface despite changes in the illumination intensity. A textbook example says that a chalk piece looks white in sunlight as it does in the moonlight. Similarly, a lump of coal looks black in both viewing conditions, although the intensity of light reflected from chalk under the moonlight is lower than the intensity of light reflected from coal under sunlight.

Using a simple reflectance model, the intensity of the light reflected from a surface (*L*_*R*_) is a product of the intensity of light incident on the surface (*L*_*I*_) and the surface albedo *S*: *L*_*R*_ = *L*_*I*_*S*. The transformation from *S* to *L*_*R*_ is a simple scaling by a constant. This scaling transformation is a group, and the simplest invariant is the ratio of the reflected light from a pair of surfaces. This simple model is the oldest model of lightness constancy, but this model cannot explain lightness constancy in natural viewing conditions with natural 3D environments (e.g., Gilchrist and Jacobsen, 1983). Explaining lightness constancy in the natural viewing conditions has to take into account the 3D shapes of objects and must be able to handle surfaces that have specular components (Adelson, 2000).

#### 2.1.4. Color Constancy

Color constancy is a generalization of lightness constancy. The human visual system has three types of cones with different absorption spectra. Surface reflectance *S*(λ) is a continuous function of wavelength λ, not just a scalar like albedo. Specifically, *S*(λ) is a % reflectance of a surface for individual wavelengths. The visible spectrum of light is usually assumed to be between 400 and 700 nm. Color constancy is crucial because it corresponds to surface reflectance, a permanent characteristic of an object. Reflectance does not change when the illuminant changes or when the object undergoes rigid motion. Achieving color constancy under these transformations (illuminant change plus rigid motion) is essential in everyday life. Humans can see the color of a fruit as the same in the morning, middle of the day, and evening, under a sunny or cloudy sky, and when looking at it from different viewing directions. The importance of rigid motion has been ignored for most of the history of the study of color constancy, although we now know that the spectrum of the reflected light depends on the surface's orientation relative to the observer. Furthermore, changing the positions and orientations of one object relative to others changes secondary reflections and introduces shadows, which, in the end, changes the spectrum of the light coming to the observer's eye.

The human vision community's effort focused on the role of illuminant, *L*(λ), which, in the general case, could be arbitrary. We know that completely arbitrary illuminants can lead to complete color constancy failures because of the ill-posedness of the inverse problem of reflectance reconstruction (inverse problems will be discussed later in this paper). However, in natural viewing conditions, daylights are well approximated by the black-body radiation, whose theory was formulated by Planck at the beginning of the twentieth century. Vision scientists realized in the 1950 and 1960s that a black body's energy spectrum is a smooth curve, which changes smoothly across the limited range of the black body temperature. This makes it possible to approximate daylights with a small number of basis functions. This allowed the use of linear models (see D'Zmura and Iverson, 1993a,b, 1994; Iverson and D'Zmura, 1994; Wandell, 1995). This was an essential step toward using group theory and their invariants in theories of color constancy, although theories of color constancy were never actually formulated as an attempt to derive group invariants. The most influential theory of this kind was von Kries's adaptation, which was formulated more than a century ago based on empirical results of color matching, well before linear models were used in theories of color vision. According to this theory, the cone absorptions are independently scaled when the same reflectance is shown under two natural illuminants (daylights) (Foster and Nascimento, 1994; Foster, 2011; Foster et al., 2016). Independent scaling could be viewed as a generalization of the ratio rule for lightness constancy. It follows that according to this rule, color constancy can be verified by computing the ratios of cone absorptions for two surface patches. Interestingly, an approximation to von Kries's law can be derived by restricting illuminants to daylights and assuming that reflectances can be represented by a linear model with three basis functions (Wandell, 1995). Despite its mathematical elegance, von Kries's law, and almost all other existing models of color constancy, are restricted to 2D surface patches and cannot handle 3D objects that undergo rigid motion (see Barron and Malik, 2015, for an example of a joint reconstruction of colors and shapes of 3D objects).

To summarize, perceptual constancies represent perceptual invariants and should always be studied in close relation to the discussion of the relevant transformation groups and the relevant permanent characteristics of objects. This has been done sometimes with shape constancy, but not with other constancies. In our view, there is no alternative to a principled approach rooted in invariants, an *approach that would connect perceptual invariants to physical invariants that represent permanent characteristics of objects and scenes*. To provide a full picture of perceptual constancies, we need to explain how the percept of the invariant physical characteristic is formed in the first place.

## 3. Noether's Theorem

Before we describe what is happening when a physical event is translated to its mental representation, as indicated in Figure 3, we will explain how physicists think about the natural law *N* in Figure 2 and how they derive conservation laws.

Physicists recognized more than a century ago that all, or almost all, natural phenomena can be explained by assuming that Nature optimizes a functional *S*, called the action^2^. Instead of applying forces to masses, and predicting the natural development of an event, which starts with known *initial conditions*, as Newton's laws work, one can start with the *boundary conditions* specifying how the event begins and ends, and explain what happens in between by assuming that nature minimizes an objective or cost function. This is how Fermat, in the middle of the seventieth century, explained reflection and refraction of light. Specifically, Fermat assumed that light minimizes the total time of travel.

We now know that nature *usually* minimizes some cost functional, in physics called the action functional, often denoted by *S*. Sometimes, however, Nature maximizes a cost functional or develops in a way that corresponds to an inflection point of the functional. Therefore, instead of talking about the least-action principle, it is more accurate to talk about the principle of stationary-action, in which an infinitesimal variation in the system's behavior leads to no changes in the cost functional. Here, we will follow the conventional name of “least-action principle.” Fermat's principle of least-time led in the eighteenth and nineteenth centuries to a full-blown theory of the least-action principle developed by Maupertuis, Euler, Lagrange, Jacobi, and Hamilton.

To discuss the relationship between the least-action principle and conservation laws, let us first present, in a simple way, how physical laws can be derived from a variational principle. Let us start with a set of coordinates, {*q*_*i*_}, *i* = 1, …, *m*, that can represent without ambiguity the position of a physical object. Examples of *q*_*i*_'s are the standard (*x, y, z*) Cartesian coordinates for a particle in a three-dimensional space, their combination with Euler angles, (*x, y, z*, α, β, γ) to describe the position of a solid, or spherical coordinates (*r*, θ, φ). But any set of variables that can be used to describe the position of a physical object uniquely would be a generalized coordinate. The trajectory of the system, which we want to describe with our physical theory, is given in terms of the generalized coordinates as a function of time *t*, i.e., *q*_*i*_ = *q*_*i*_(*t*). The goal of a physical theory is to find the functions *q*_*i*_(*t*) that correctly represent the system's actual trajectory as it evolves with time. This is done with the variational principle that leads to the Euler-Lagrange equation, which was one of the most fundamental achievements of mathematical physics of the eighteenth century. Appendix B in Supplementary Material shows all the important steps of this derivation to allow the reader to appreciate its generality. In the text below we will only state the problem and its solution in the form of Euler-Lagrange equation (for a complete presentation of a least-action principle, we recommend Arnold, 1989 or Goldstein, 1980).

Let *L* be a function that depends on *q*_*i*_(*t*), ${\dot{q}}_{i}\left(t\right)$, where ${\dot{q}}_{i}\left(t\right)=dq_{i}\left(t\right)/dt$, and *t*, i.e. $L=L\left(q_{i}\left(t\right),{\dot{q}}_{i}\left(t\right),t\right)$. *L* is the Lagrangian function, and for some systems it is written as the difference between the kinetic and potential energies, i.e.,

L=K-V,

where *K* is the kinetic energy and *V* is the potential energy. Let us define *S* as the integral in time of *L*, i.e.,

(1) $$\begin{array}{l} S\left[q_{i}\left(t\right),{\dot{q}}_{i}\left(t\right),t\right]=\int_{t_{1}}^{t_{2}}L\left(q_{i}\left(t\right),{\dot{q}}_{i}\left(t\right),t\right)dt. \end{array}$$

The principle of least action states that if *q*_*i*_(*t*) is the actual solution to the motion of the system, then small changes to *q*_*i*_(*t*) → *q*_*i*_(*t*) + δ*q*_*i*_(*t*) result in no changes to *S*, i.e.,

(2) $$\begin{array}{l} \delta S=\delta \left[\int_{t_{1}}^{t_{2}}L\left(q_{i}\left(t\right),{\dot{q}}_{i}\left(t\right),t\right)dt\right]=0, \end{array}$$

since *S* is in a minimum (or maximum or saddle point).

We will not show all the details of how to find the equations of motion, but instead, we will sketch its derivation from the variational principle. We start with an action *S* as defined in Equation (1), and assume that the functions *q*_*i*_(*t*) are the actual trajectories of the particle for any time *t*. We then make small perturbations to *q*_*i*_(*t*) → *q*_*i*_(*t*) + δ*q*_*i*_(*t*), with the additional constraint that δ*q*_*i*_(*t*_1_) = δ*q*_*i*_(*t*_2_) = 0. The idea is that we are taking two arbitrary points along the actual trajectory of a particle, namely *q*_*i*_(*t*_1_) and *q*_*i*_(*t*_2_), and then considering all possible trajectories between those two points that differ from the actual one by a perturbation δ*q*_*i*_(*t*). This perturbation is assumed to be a twice-differentiable arbitrary function. After an integration by parts, as shown in Appendix B in Supplementary Material, we arrive at the following equations:

(3) $$\begin{array}{l} \frac{\partial L}{\partial q_{i}}-\frac{d}{dt}\left(\frac{\partial L}{\partial {\dot{q}}_{i}}\right)=0. \end{array}$$

Equations (3) are known as Euler-Lagrange (EL) equations, and their solution minimizes the action *S* between arbitrary times *t*_1_ and *t*_2_. EL equations are significant not only because they render a solution to the variational problem, but also because they provide a generalized framework for dealing with dynamical systems in mechanics. They also allow one to illustrate how, for some simple cases, Emmy Noether established a mathematical link between symmetries of natural laws and conservation laws. One remarkable aspect of Noether's fundamental contribution to physics is that it was a mathematical theorem. It is important to point out that EL equations were derived under quite general assumptions about the action *S* which is minimized by a trajectory in some space. It is this generality of the mathematical formulation that allowed researchers to apply a least-action principle and EL equations to inverse problems in computational vision (Foster, 1978; Ben-Yosef and Ben-Shahar, 2012).

As an example of how EL are solutions to the mechanical problem of motion, let us examine the one-dimensional case of a particle of mass *m* subject to a potential *V*(*x*), where *x* is the Cartesian coordinate. The Lagrangian for this particle is given by

(4) $$\begin{array}{l} L=K-V=\frac{1}{2}m{ẋ}^{2}-V\left(x\right). \end{array}$$

Substituting (4) into (3), we obtain

(5) $$\begin{array}{l} \frac{\partial L}{\partial x}-\frac{d}{dt}\left(\frac{\partial L}{\partial ẋ}\right)=\frac{\partial V}{\partial x}+mẍ=0, \end{array}$$

where

$$ẍ=\frac{dẋ}{dt}=\frac{d^{2}x}{dt^{2}}=a.$$

Since *a* is the acceleration of the particle in the direction *x*, Equation (5) is equivalent to Newton's Second Law of motion, as

$$-\frac{\partial V}{\partial x}\equiv F_{x}.$$

So, the least-action principle, with an appropriate Lagrangian, can be used to derive Newtonian mechanics. According to it, the actual trajectories followed by a particle are the ones that minimize the time integral of the quantity *L* = *K* − *V*, i.e., it minimizes

S=∫Ldt.

However, the least-action principle is much more general than Newtonian mechanics, whose limited domain are point particles. It can be applied not only to systems of particles, but also to much more complicated systems, such as constrained systems and classical physical systems with infinite degrees of freedom, such as fluids, Maxwell's electromagnetic theory, gauge theories, and Einstein's theory of gravitation. It can even be used to derive Schroedinger's equation in quantum mechanics, which is related to the eikonal equation in optics. In the present paper we are applying a least-action principle to inverse problems in vision, in general, and to 3D shape perception in particular. But not all physics can be obtained from a variational principle. In fact, though the physical theories that cannot be derived from the least action principle are in the minority, they constitute important cases. One example of such theories is statistical mechanics, as the ergodic hypothesis, an essential assumption for Boltzmann's H-theorem does not seem to be derivable through a variational principle.

From the variational principle, Noether proved an important theorem that relates symmetries to conserved quantities. Noether's first theorem states that every differentiable symmetry of the action of a physical system with conservative forces has a corresponding conservation law. Proving it in the general case would go beyond this paper's scope, but we are now ready to provide some elementary examples illustrating her theorem of conservation laws from symmetries. Consider first the case when *L* does not depend on *q*_*i*_, which means that any transformation *q*_*i*_ → *q*_*i*_ + δ*q*_*i*_ leaves *S* invariant. To see the conserved quantity, let us take the more familiar three-dimensional Cartesian coordinates describing a single particle. Then, for each particle coordinates *x*, *y*, and *z*, there is a corresponding EL equation, similar to Equation (5). When *L* is independent of, say, *x*, it follows that the EL for *x* reduces to

$$-\frac{d}{dt}\left(\frac{\partial L}{\partial ẋ}\right)=0$$

or

$$\frac{\partial L}{\partial ẋ}=\text{constant}.$$

This is a conservation law: the partial derivative of *L* with respect to ẋ is conserved in time. In the case of a particle, where the term in *L* depending on ẋ is the kinetic energy, i.e., for ẋ it is $\frac{1}{2}m{ẋ}^{2}$, it follows that the independence of *L* with respect to *x* leads to the conservation of

$$\frac{\partial L}{\partial ẋ}=\frac{\partial }{\partial ẋ}\left(\frac{1}{2}m{ẋ}^{2}\right)=mẋ\equiv mv_{x},$$

where *v*_*x*_ is the component of the velocity in the direction *x*. In other words, the invariance (symmetry) of *L* with respect to *x* leads to the conservation of momentum *p*_*x*_, defined as *mv*_*x*_, in the direction *x*. We say that spatial position and linear momentum are conjugate variables. Similar results can be obtained if we use, instead of Cartesian coordinates, spherical coordinates. However, in the case of spherical coordinates, the invariance with respect to angular coordinates leads to the conservation of angular momentum. So, the angular position and angular momentum are conjugate variables, too.

The Euler-Lagrange equation allows us to derive, straightforwardly, an invariant quantity when the Lagrangian $L\left(q_{i},{\dot{q}}_{i}\right)$ is independent of *q*_*i*_ or its time derivatives, ${\dot{q}}_{i}$. However, other symmetries are not as directly obtainable as, e.g., the conservation of momentum above. An example is the conservation of energy, whose derivation, in the spirit of Noether's theorem, is presented in Appendix C in Supplementary Material.

However, Noether's theorem is much more general. It states that each symmetry of the Lagrangian under a continuous transformation (such as time translation) corresponds to a conservation law. In the above case, a symmetry under time translation results in the conservation of energy. For a mechanical system, symmetries under spatial translation correspond to the conservation of momentum, and rotational symmetry to angular momentum. See Hanc et al. (2004) for a complete derivation, Lemons (1997) for a presentation of a least-action principle, including its history, and Morton Tavel's English translation of Noether's original 1918 paper, in which she proved her theorem using variational calculus, in Noether (1971). Note that a conservation law is an invariant—it can be thought of as a higher-order invariant compared to the symmetry from which it is derived. The variable to which a natural law is invariant and the corresponding conservation quantity are called conjugate variables in physics. And so, time and energy, position and momentum, angular position and angular momentum are such pairs of conjugate variables.

If symmetries and conservations both refer to invariance, what is the difference between them? In a nutshell, conservation laws refer to invariance in time, whereas symmetries can refer to invariance to other coordinates or parameters. For example, the conservation of momentum means that system's total momentum is the same at two different times. This conservation of momentum is associated with another symmetry: the invariance of the Lagrangian to spatial translations. However, the conservation of momentum is itself a symmetry since the momentum is invariant under time translation. It is just a physics convention to reserve the word *conservation* to quantities that are constant in time. Another way to see the difference between symmetries and conservations is to realize that they play a very different role in Noether's theorem. Specifically, conservations are derived from symmetries. The symmetry of a Natural Law N means that the Law is invariant under a transformation of the coordinate system (spatial or temporal), as illustrated by the entire diagram in Figure 2. Conservation, on the other hand, refers to a quantity that stays invariant when an initial state *u* [or Θ(*u*)] evolves with time into the final state *N*(*u*) [or *NΘ*(*u*)] as shown in Figure 2.

In section 4, we will remind the reader how a simplicity principle has been used in perception. It has been commonly agreed that simplicity principle in perception is entirely analogous to the least-action principle in physics. This was first conjectured by Mach (1914), formally implemented for the first time by Foster (1978), and adopted by the computer vision community a decade later by Poggio and Koch (1985). With two cornerstones of physics, symmetry and least-action, already used in theories of perception, the time is to find the place for conservation laws in theories of perception and for conjugate variables in perception. Section 5 will show that mental representations of abstract characteristics of physical objects are such conservations.

## 4. Perception Viewed as an Inverse Problem

This section will describe a computational formalism that is commonly used to explain how a 3D percept is produced from a 2D retinal or camera image. The physical environment is 3D, and our percept of the environment is 3D. But the visual data that come from the retinal image are 2D. It follows that the problem of inferring 3D interpretation from a 2D image is an ill-posed inverse problem (Poggio et al., 1985; Pizlo, 2001, 2019). By ill-posed, we mean here that a 2D image does not specify a unique 3D interpretation. This is an intuitively obvious observation, but it leads to important mathematical implications. A perspective projection from a 3D space to a 2D image is described by the rules of optics, but this transformation is not a group, and it does not have invariants. In particular, there is no unique inverse perspective projection from 2D to 3D. The other three axioms of a group are also violated in this case. It follows that without additional assumptions, a 2D perspective image does not allow the computation of any 3D invariants, including the 3D shape of an object. We must first convert an ill-posed problem into a well-posed one (Pizlo, 2019). The only known method of converting an ill-posed inverse problem into a well-posed one is to impose constraints on the family of possible interpretations. If these constraints are effective, the interpretation will be unique and veridical. Once this happens, our transformation between a 3D space and a 2D image will become a group and will allow the computations of 3D invariants from a 2D image. Poggio et al. (1985) provided a list of several “early vision” functions that should be treated as ill-posed inverse problems: edge detection, optical flow, 3D surface reconstruction, spatiotemporal approximation, color reconstruction, shape from shading, binocular vision. One can add several other functions, such as lightness perception, shape recovery, figure-ground organization. In fact, any interpretation of sensory data is an inverse problem and must be treated as such. Combining visual data with a priori constraints takes the form of a cost functional, where the 3D interpretation corresponds to the minimum of the functional.

We will focus on 3D shape recovery from a 2D perspective image. Most natural objects are approximately symmetrical: animal bodies, plants, and even human-made objects are symmetrical. Mirror-symmetry is the most common type of symmetry characterizing objects: bodies of almost all animals are mirror-symmetrical because of locomotion, and many human-made objects are mirror-symmetrical, too. Real objects are never perfectly symmetrical, but they are nearly so. When we say that an object is symmetrical, we refer to the redundancy inherent in the object. In a mirror-symmetrical object, the two halves are identical. This is a particular case of the reflection group, where a reflection with respect to the symmetry plane is the identity transformation. The reflection group is the simplest: it consists of identity and reflection, which is its own inverse. The reflection group does not care whether objects are characterized by reflection symmetry. The case of mirror-symmetrical objects is therefore special. Such objects can be described as (i) *invariant* under mirror-reflection, and as (ii) being geometrically *redundant* because the two symmetrical halves are identical. These two different, but closely related, aspects of symmetry are essential in producing 3D veridical percepts from 2D images. The invariance of an object under reflection means that parity (chirality, handedness) of the object does not appear in its representations. This also means that parity is absent from the cost functional used to recover 3D shapes from 2D images. The absence of parity will become important in the next section when discussing the conservations implied by minimizing the cost functional. The redundancy aspect of symmetrical objects is used as an *a priori* constraint in the cost functional. It is our belief that redundancy is the most effective constraint in solving ill-posed inverse problems.

**Figure 4:**
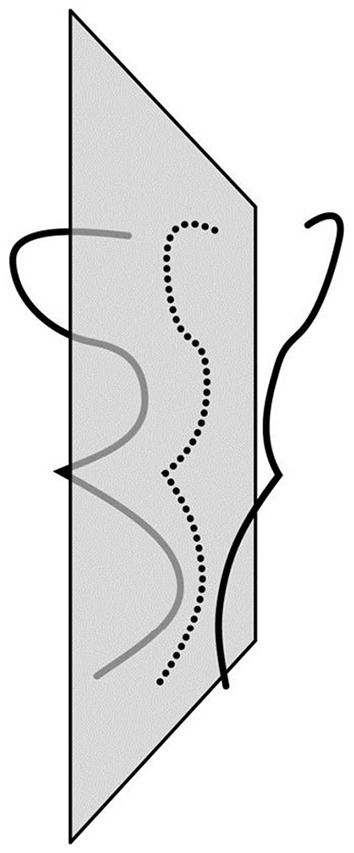
Illustration of the path (dotted line) formed in a plane by connecting midpoints of pairs of mirror-symmetrical points.

The following equation is a generic formulation of a cost function used to recover a 3D mirror-symmetrical shape from a single 2D perspective image,

(6) $$\begin{array}{l} E\left(X\right)=\|A\left(X\right)-Y\|+\lambda \|P\left(X\right)\|, \end{array}$$

where the norms represent integrals along a path, *A*(*X*) is a perspective projection of a 3D shape *X* onto the 2D image, *Y* is a given 2D image, *P*(*X*) compares the two halves of *X* and λ is a weight representing the reliability of the visual data relative to the reliability of the constraint. Unlike the least-action principle in mechanics, where the integration is along a path that develops in time, in 3D shape perception, the process develops along a spatial path. In 3D shape recovery, the path is a planar curve connecting midpoints of pairs of mirror-symmetrical points. We know that this path is a planar curve because this path lies on the symmetry plane of the object (see Figure 4 for an example of such path). So, in mechanics, a trajectory of an object moving in a gravitational field minimizes mechanical action. In 3D shape recovery, the planar curve connecting midpoints of pairs of symmetrical points of an object minimizes cost functional *E*. What this last sentence means is that the recovery of a 3D mirror-symmetrical shape is mathematically equivalent (isomorphic) to the computation of the planar curve in 3D space connecting midpoints of pairs of symmetrical points. 3D shape recovery has never been formulated that way, although this fact was implicitly present in the derivation provided by Sawada et al. (2011). It follows that one can treat the parameter representing the movement along the 3D curve connecting midpoints as time, making 3D shape recovery mathematically identical with Hamiltonian mechanics.

**Figure 5:**
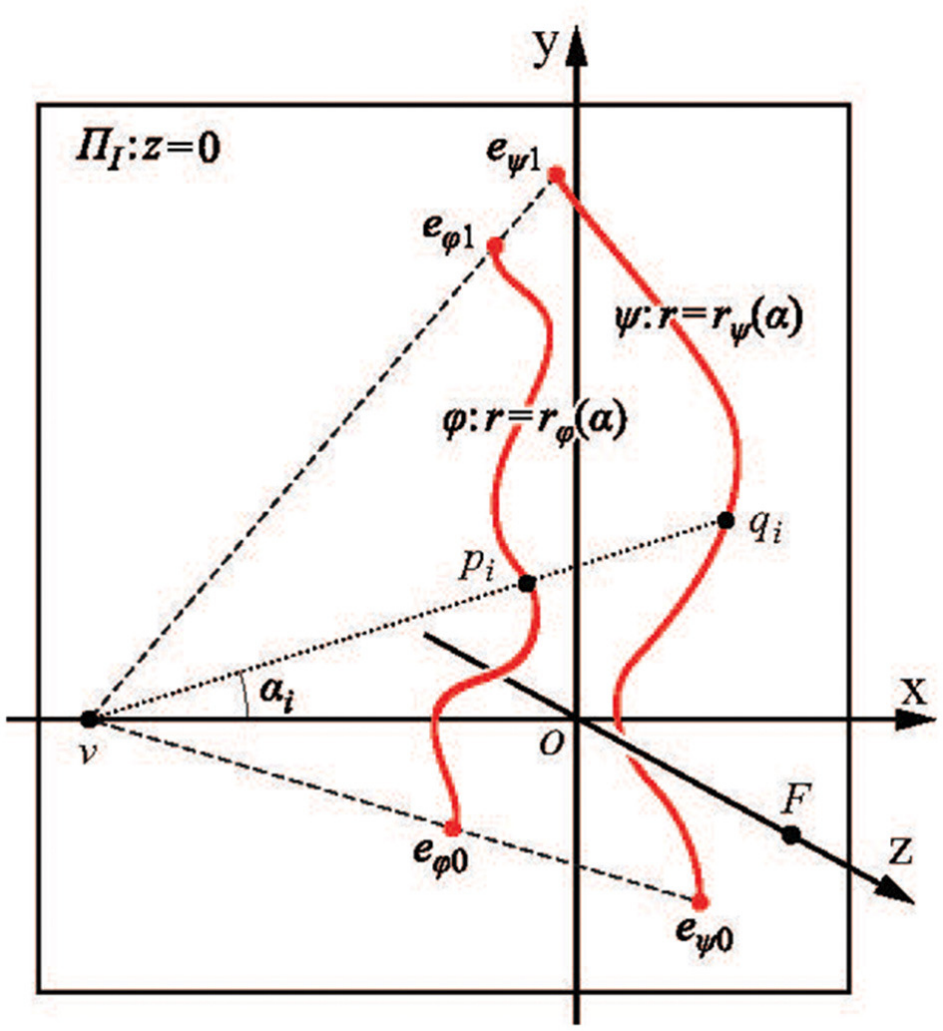
The two curves, φ and ψ, are 2D perspective images of a pair of 3D mirror-symmetrical curves Φ and Ψ that are reconstructed from this single 2D image. *F* is the center of perspective projection, and its coordinates are (0, 0, *z*_*F*_). The principal point is at coordinates (0, 0, 0) (Sawada et al., 2011).

Below are the key steps from Sawada et al. (2011) illustrating how this is done. Figure 5 shows the notation used. The choice of the coordinate system in Figure 5, with the origin at the principal point, is not commonly used. Sawada et al. adopted it to illustrate how a 3D recovery from a 2D orthographic image (the case widely used in the literature) is a limiting case of recovery from a perspective image when *z*_*F*_ goes to infinity. The curves φ and ψ are described in a polar coordinate system (*r*, α), with the center at the vanishing point *v* = (*x*_*v*_, 0, 0) representing the symmetry plane of Φ and Ψ. Symmetry correspondence of a point *p*_*i*_ on φ and *q*_*i*_ on ψ is established by lines emanating from the vanishing point *v*. The 2D points *p*_*i*_ and *q*_*i*_ are perspective images of 3D points *P*_*i*_ and *Q*_*i*_.

The symmetry plane of Φ and Ψ is as follows:

$$-\frac{x_{v}}{z_{F}}x+z+\frac{d}{c}=0.$$

The ratio $\frac{-d}{c}$ is the intersection of the symmetry plane and the z-axis. The vanishing line *h* of the symmetry plane has the following equation on the image plane:

$$x_{h}=-\frac{z_{F}^{2}}{x_{v}}.$$

A perspective projection *m*_*i*_ of the midpoint *M*_*i*_ of *P*_*i*_ and *Q*_*i*_ can be computed as follows:

(7) $$\begin{array}{l} m_{i}={\left[\begin{array}{c} x_{m_{i}}\\ y_{m_{i}}\\ 0 \end{array}\right]}^{T}={\left[\begin{array}{c} x_{v}+\frac{2r_{\varphi }\left(\alpha_{i}\right)r_{\psi }\left(\alpha_{i}\right)}{r_{\varphi }\left(\alpha_{i}\right)+r_{\psi }\left(\alpha_{i}\right)}\cos\alpha_{i}\\ \frac{2r_{\varphi }\left(\alpha_{i}\right)r_{\psi }\left(\alpha_{i}\right)}{r_{\varphi }\left(\alpha_{i}\right)+r_{\psi }\left(\alpha_{i}\right)}\sin\alpha_{i}\\ 0 \end{array}\right]}^{T}. \end{array}$$

Note that *m*_*i*_ is not a midpoint of *p*_*i*_ and *q*_*i*_, but it is clear from Equation (7) that *m*_*i*_ can be computed directly from the image points *p*_*i*_ and *q*_*i*_. The final step is the computation of the *z*-coordinates of points on Φ and Ψ, namely

(8) $$\begin{array}{l} z_{\Phi_{i}}=z_{F}+\frac{2r_{\psi }\left(\alpha_{i}\right)\left(z_{F}+\frac{d}{c}\right)x_{h}}{\left(r_{\varphi }\left(\alpha_{i}\right)+r_{\psi }\left(\alpha_{i}\right)\right)\left(x_{m_{i}}-x_{h}\right)} \end{array}$$

and

(9) $$\begin{array}{l} z_{{\text{Ψ}}_{i}}=z_{F}+\frac{2r_{\varphi }\left(\alpha_{i}\right)\left(z_{F}+\frac{d}{c}\right)x_{h}}{\left(r_{\varphi }\left(\alpha_{i}\right)+r_{\psi }\left(\alpha_{i}\right)\right)\left(x_{m_{i}}-x_{h}\right)}. \end{array}$$

The *X* and *Y* coordinates of points on Φ and Ψ are computed from parametric equations of the projecting lines emanating from *F* and intersecting the image plane at *p*_*i*_ and *q*_*i*_ (see Sawada et al. for details).

Equations (8), (9) can be substantially simplified using a different parametrization (see Pizlo, 2019). But the parametrization used by Sawada et al. is particularly useful in our context because it shows how 3D recovery based on symmetry constraint is produced from a curve connecting images of midpoints.

If the object *X* is mirror-symmetrical, 3D mirror reflection with respect to the symmetry plane leaves the object invariant. It should be clear that the cost functional E is also invariant to mirror reflection of *X*. This invariance to mirror reflection is analogous to the invariance of action *S* to such transformations as translations in space and time. The main difference is that physical translations in space and time are continuous transformations, whereas mirror reflection is a discrete transformation.

All visual inverse problems are formulated by using a cost function like that in Equation (6). The only difference across visual functions is how the visual data is defined and what the constraints are. The cost functional *E* is sometimes referred to as energy, but in all computational models that solve inverse problems, the use of constraints is referred to as the operation of a simplicity principle. This is how Foster (1975, 1978) solved apparent motion problem, and how Poggio et al. (1985) described the solution of early vision problems. Both Foster and Poggio pointed out the remarkable similarity between the simplicity principle and a least-action principle. It should be obvious, however, that perception is different from mechanics. The laws of physics (i) do not perform inferences based on incomplete data, and (ii) there are no sensory data in physics. But once the cost functional is set up in the visual system of the brain, the neurons are likely to minimize the cost functional through chemical or electrical interactions (Poggio and Koch, 1985). This way, the least-action principle of physics (and chemistry) acts like a piece of computing machinery that solves an ill-posed inverse problem of perception—(see Horn and Schunck, 1981; Weiss et al., 2002; Stocker, 2006; Dold et al., 2019; Greydanus et al., 2019; Lutter et al., 2019). It is important to point out that once a least-action has been adopted in models of vision and cognition, a few authors have already made the next step and used Noether's theorem to explain vision (Weiss, 1997) and motor control (Huh and Sejnowski, 2016).

There is a probabilistic version of the cost function used to solve ill-posed inverse problems in perception. If constraints are probabilistic priors, they can be combined with the probabilistic sensory data using Bayesian inference. This can certainly be done. The first term on the righthand side of the cost function in Equation (6) evaluates how far is the particular 3D interpretation from the 2D sensory data. If the norm is quadratic, one can think of a Gaussian pdf evaluating this difference. Smaller differences correspond to the higher likelihoods. The second term on the righthand side of the cost function evaluates the departure from the perfect symmetry interpretation. This can be represented by a Gaussian pdf, as well. After the two probabilities are multiplied, we obtain a Bayesian posterior. And indeed, under some fairly general assumptions, the minimum of the cost function corresponds to the maximum a posteriori (MAP) estimate. In our cost function, the parameter lambda is implicitly represented in the Bayesian formula by the ratio of variances of the likelihood function and the prior. If these variances can be independently estimated, the choice of the parameter lambda in the cost function is no longer *ad hoc*. The Bayesian formulation was used throughout the book titled “Perception as Bayesian Inference” and edited by Knill and Richards (1996). Chater's (1996) paper discussed the equivalence of the two formulations: probabilistic and deterministic (see also Poggio et al., 1985; Pizlo, 2001). One of us has used both formulations (compare Li et al., 2009, 2011; Jayadevan et al., 2018). One clear advantage that Bayesian formalism offers is that it naturally allows for updating the priors, if updating priors is empirically justified.

## 5. Mental Representations as Conservations

The cost functional E in 3D shape recovery corresponds to action *S* in physics. As pointed out in section 4, our cost functional is invariant (symmetric) to mirror-reflection of the object because the mirror-reflection of a mirror-symmetrical object is an identity transformation. In other words, *E* does not depend on the parity of the object. This suggests, following the logic of Noether's theorem, that there must be a conserved characteristic (variable) implied by this invariance and derivable from the minimum of *E* (Pizlo, 2019). In other words, presumably there is a conjugate variable corresponding to the object's parity, the same way position and linear momentum, angular orientation and angular momentum, and time and energy are conjugate variables^3^. What is this conserved characteristic in the case of perception of 3D symmetrical objects?

We want to point out that we use symmetry twice in our 3D shape perception theory, but what we are describing is not a circular argument because we use two different aspects of symmetry: redundancy and invariance. First, the simplicity constraint *P*(*X*) in the cost functional verifies the degree of redundancy of a mirror-symmetrical object, and, second, our cost functional, itself, is symmetric (invariant) to mirror-reflection of the object. We know, based on theorems of projective geometry, as well as on numerous computational tests, that the minimum of the cost functional *E* corresponds to veridical (correct) 3D shape recovery, where by shape we mean an invariant of rigid motion plus uniform size scaling (Li et al., 2011; Sawada et al., 2011; Michaux et al., 2016, 2017; Jayadevan et al., 2018). In the absence of noise and uncertainty, minimizing the cost function *E* leads to closed-form formulas (Equations 8, 9) for 3D shape recovery that can be thought of as being analogous to the EL equation in mechanics. Specifically, the minimum of the integral along the entire path connecting midpoints of pairs of symmetric points is mathematically equivalent to a spatially local operation performed one pair of points a time.

Is this theory empirically testable? The answer is yes: such a test was performed by the first author (Li and Pizlo, 2011). Li and Pizlo (2011) used a shape constancy task in which the subject was asked to discriminate whether two successively presented 3D stimuli had identical shapes (i.e., whether they were identical up to a 3D rigid motion and size scaling). When the 3D shapes were identical, they were shown from viewing directions that were 90 deg apart. So, this experiment tested shape perception as a perceptual invariant (see Figure 3).

**Figure 6:**
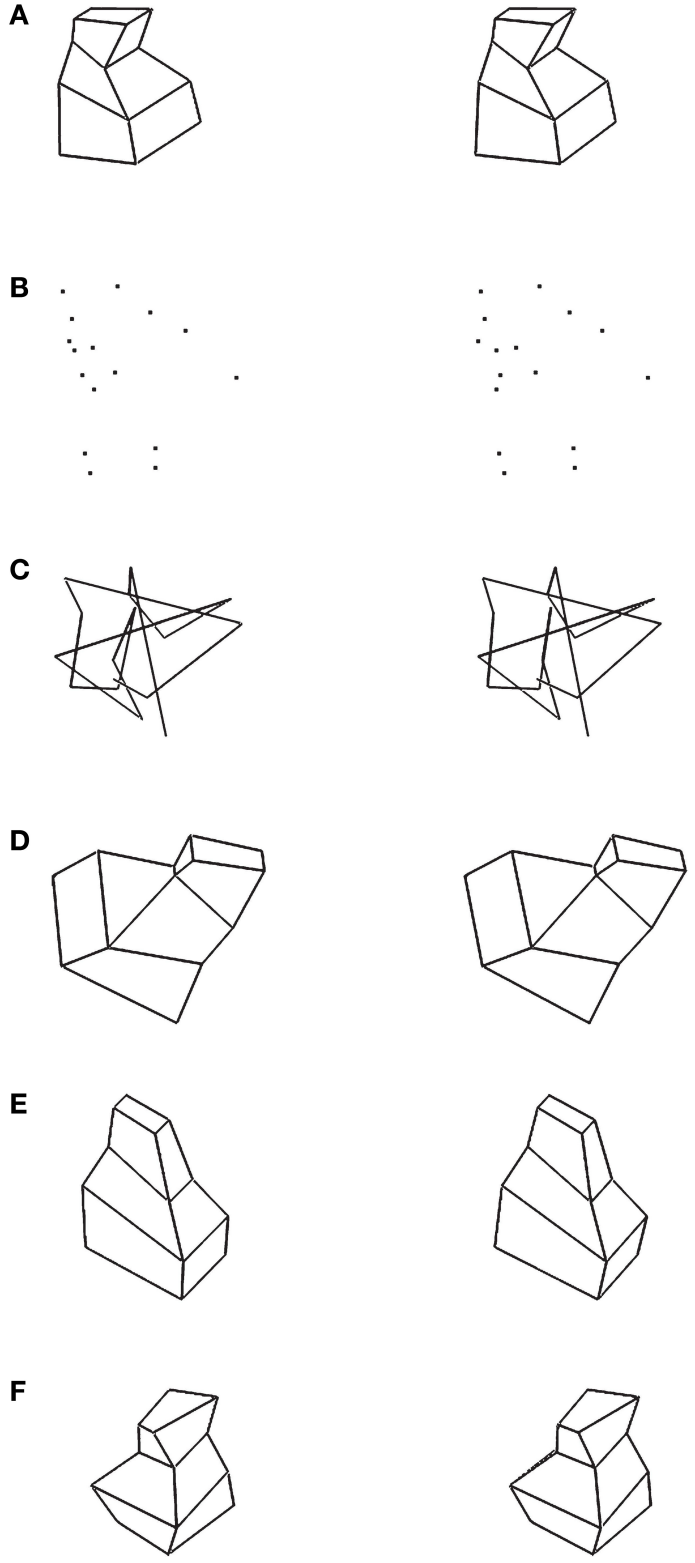
Stereoscopic images (crossed fusion) of the six types of stimuli used in Li and Pizlo (2011) study. **(A)** Polyhedron with one symmetry plane and planar surfaces. **(B)** 16 vertices which were obtained by removing the edges from the stimulus of type **(A)**. **(C)** Polygonal line. The 16 vertices were connected randomly. **(D)** Partially non-planar and symmetric polyhedron. **(E)** Planar and asymmetric polyhedron. **(F)** Non-planar and asymmetric polyhedron.

Six types of 3D objects with different constraints were used (see Figure 6). Stimulus (Figure 6A) was a mirror-symmetrical polyhedron. Stimulus (Figure 6B) (vertices) was generated by removing all edges in stimulus (Figure 6A) and showing only vertices. Stimulus (Figure 6C) (polygonal line) was generated by randomly connecting the vertices in stimulus (Figure 6B). These three types of stimuli were generated using the same method; the differences were related to how (if at all) the vertices were connected. Stimulus (Figure 6D) was a symmetric and partially non-planar polyhedron. It had a plane of symmetry, but its six lateral surfaces were not planar. Stimulus (Figure 6E) was a planar and asymmetric polyhedron. It was generated by taking a half of a stimulus (Figure 6A). Stimulus (Figure 6F) was a non-planar and asymmetric polyhedron. For each type of stimuli, there were two viewing conditions: the 3D shapes were viewed binocularly (disparity condition) or monocularly (no disparity). The stereoscopic images of these six types of stimuli are shown in Figure 6.

**Figure 7:**
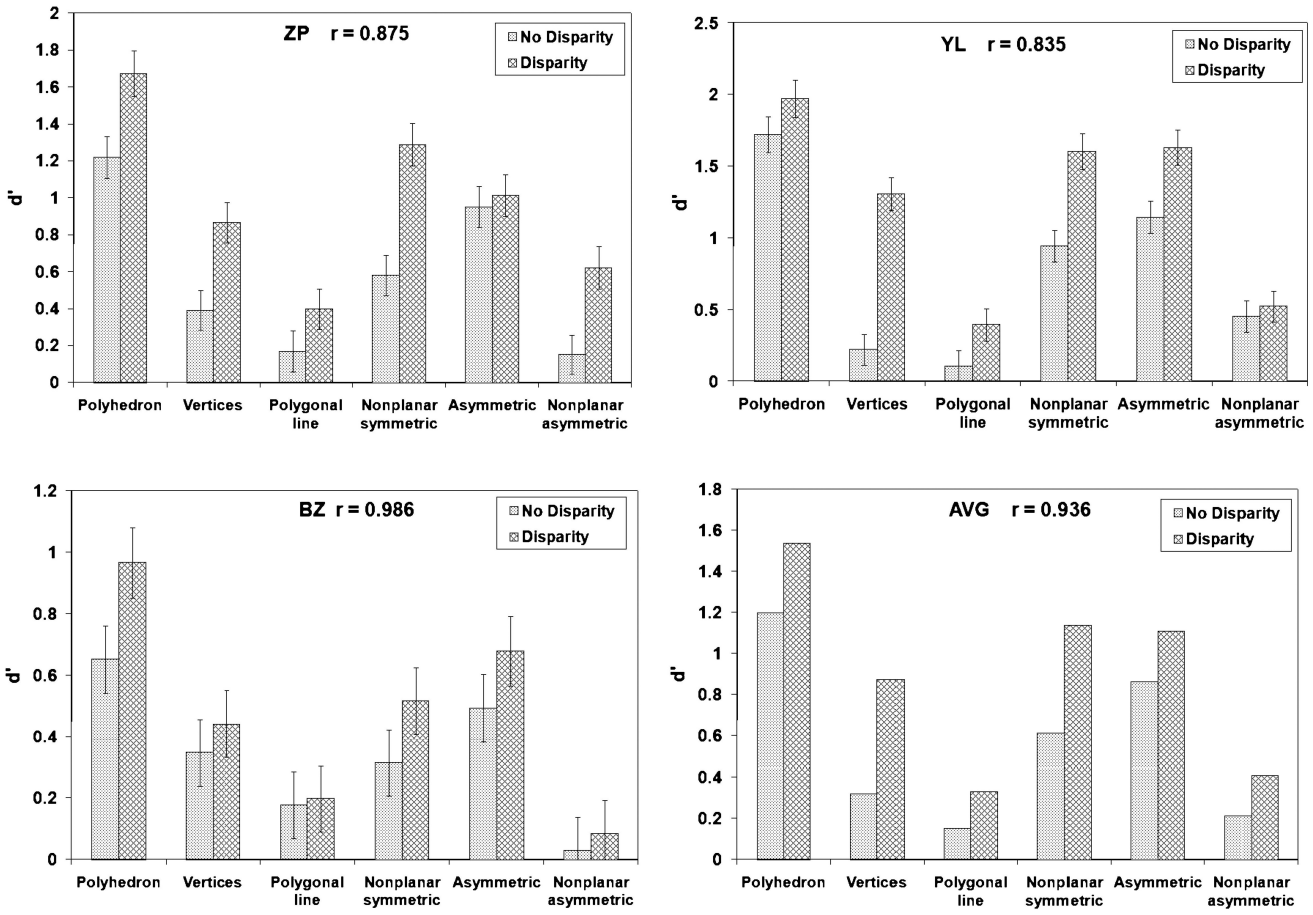
Three subjects' performance and the averaged performance (d') across the six types of objects in Li and Pizlo (2011) study.

If, contrary to our theory, perceptual 3D shape recovery is not based on the constraint of symmetry of objects, one expects binocular performance to be equally reliable across all 6 types of stimuli. At the same time, monocular performance should be at chance level for all 6 types of stimuli. Binocular performance is expected to be better than monocular performance because binocular disparity is known to be an effective depth cue. If, on the other hand, perceptual 3D shape recovery is based on the constraint of symmetry of objects, as predicted by our theory, both monocular and binocular performance should be better with symmetrical stimuli than with asymmetrical ones. Figure 7 shows the three subjects' performance (d'), as well as their averaged performance. The discriminability measure d' represents the detection ability in Signal Detection Theory. It is estimated from hits and false alarms for the two types of trials, same vs. different shapes. Chance performance is represented by d' = 0. Higher d' represent better performance. We focus here on comparing performance in the “polyhedron” and “polygonal line” conditions: polyhedron stimulus was symmetrical and polygonal line stimulus had no trace of symmetry. The vertices of these two types of stimuli were generated the same way and the only difference was how the vertices were connected. Performance with the symmetrical polyhedron was reliable (d' significantly greater than zero) with both monocular and binocular viewing, while performance with polygonal lines was close to chance level with both monocular and binocular viewing. These results provide support to the claim that the visual system uses the geometrical theorem describing how a 3D symmetrical shape can be recovered from a 2D image. These results do not prove that the visual system uses the actual computations described in this theorem, but they clearly show that 3D symmetry is essential in shape constancy.

There are two implications of our theory. First, we started with a mirror-symmetrical property of an object, which refers to a reflection group in which there is only one transformation, identity. After we applied the simplicity (least-action) principle, we recovered a 3D shape, which is invariant under a larger group of transformations, called similarity. As pointed out in the previous paragraph, the similarity group includes rigid translations, rigid rotations, reflections, and uniform size scaling. The fact that a symmetry group never gets smaller as things develop in Nature is called symmetry principle and has been established by Pierre Curie in 1894. Specifically, what this principle says is that the symmetries of the cause are always present in the effect. In our case, the 3D physical symmetrical object is the cause, and the 3D mental representation of the shape of the object is the effect. As a result, 3D mirror symmetry allows the observer to identify individual 3D objects in the environment just by looking at them, a step that has conventionally been called a figure-ground organization (Michaux et al., 2016). Second, the fact that our cost functional (action) is symmetric (invariant) to some transformation (here, reflection), implies a conservation law, by an argument analogous to Noether's theorem. Indeed, a veridical mental representation of a 3D shape of a physical object is a conservation of the mapping from the physical world to mental—see Figure 3. When we look at a wooden chair, our mental representation is characterized by the same 3D shape as the shape of the chair. But our mental representation of a wooden chair is not made of wood. So, many, if not most, of the chair's physical characteristics are lost during the transformation between the physical world and mental. But not everything is lost. The 3D shape is invariant (conserved). This is similar to the situation when two cars collide. After the collision, the vehicles are damaged or destroyed. So, cars are not invariant. But the total linear momentum is invariant.

From this second implication, we conclude that the parity of a 3D symmetrical object and its 3D perceived shape are conjugate variables the same way spatial position and linear momentum are conjugate variables. So, the claim that symmetry is the sine qua non of shape, a conjecture stated by one of us Li et al. (2013), now has a deeper mathematical meaning that is a consequence of Noether's theorem. This means that if an object has no trace of symmetry, its shape cannot be perceived. When depth cues, such as binocular disparity, motion parallax, texture or shading are available, a 3D object will be perceived as 3D, but its 3D shape will not be recovered by the visual system and shape constancy will fail. This fact has been demonstrated repeatedly, starting with Rock's experiments. Multiple papers reported the same result, namely, that with amorphous wire objects and random star shapes that have no trace of symmetry, shape constancy completely fails (Rock et al., 1981, 1989; Rock and DiVita, 1987; Edelman and Bülthoff, 1992; Farah et al., 1994; Pizlo and Stevenson, 1999; Chan et al., 2006; Li and Pizlo, 2011). Rock and his colleagues thought that their result applied to all objects. It does not. When an object is mirror-symmetrical, shape constancy is perfect, or nearly so (Pizlo and Stevenson, 1999; Chan et al., 2006; Li and Pizlo, 2011; Li et al., 2011; Jayadevan et al., 2018).

## 6. Summary and Directions for Future Research

Our paper's title is a slight modification of Ernst Cassirer's seminal 1944 paper's title (Cassirer, 1944). Cassirer emphasized the role of group invariants in explaining perceptual constancy, but he missed the point that a 3D–2D perspective projection is not, in the general case, a group and does not have invariants. Cassirer was not a psychologist, and he did not perform any experiments on perception that could verify his conjectures. He was also apparently unaware of the fundamental relevance of invariance in physics, including Noether's paper. J.J. Gibson picked up the invariance concept in his 1950 book (Gibson cited Cassirer's article). Gibson, a psychologist, built his career around the concept of invariance, introducing his theory of “direct perception.” However, Gibson's theory of direct perception, in which he assumed that the 3D interpretations are available in the 2D retinal image and that no computations are needed, never went beyond verbal statements. Nevertheless, Gibson's emphasis on dealing with 3D stimuli did have a lasting impact, and his theory of direct perception was modified by Marr's group to became a theory of 3D reconstruction. This included the use of motion, texture gradient, as well as projective invariants. We are in an excellent position to extend and reformulate Cassirer's and Gibson's ideas because we are writing 75 years after Cassirer published his paper and 40 years after Gibson published his last book. We extended Cassirer's observations by showing how the invariance and redundancy aspects of symmetry lead to a theory that explains veridical 3D shape perception. In our approach, a 3D visual representation of the physical environment results from solving an ill-posed inverse problem. Contrary to Gibson's claim, the 3D veridical representation is not “picked up” from the retinal image or the environment.

Most importantly, we established, for the first time, a close connection between the mathematical formalism of 3D shape perception and the formalism used in Physics. This allowed us to explain perceptual constancy as a conservation law resulting from applying a simplicity principle to the symmetries of physical objects. Finally, formulating perception in the context of group invariants proved to have important implications for how experiments on perceptual constancies should be designed. Since perceptual constancies refer to permanent characteristics of objects, which themselves are group invariants, a perceptual constancy experiment must employ the entire relevant group. This has rarely been done, which probably explains the multiple confusions and contradictory results that permeate the subject's history.

We conclude by discussing several implications of our theory for future research, both empirical and theoretical.

First, consider the area called intuitive physics, which refers to the fact that humans, including infants, understand many physics concepts and phenomena without ever taking physics classes (Spelke, 1990; Spelke et al., 1994; Spelke and Kinzler, 2007). The fact that the visual system has at least qualitative knowledge about the three pillars of Physics, symmetry, least-action, and conservation, suggests that the phenomenon called “intuitive physics” resides in the core of the visual system, rather than at later stages of cognitive processing. But note that contrary to much of the existing literature on intuitive physics, we emphasize the concepts included in Noether's theorem rather than Newton's concepts. The main difference between the two is that Newton's formulation is vectorial, whereas Noether's work is grounded on analytical mechanics. Furthermore, Noether's theorem links the least-action principle to symmetries and conservation laws. This connection between those three fundamental concepts did not exist before Noether proved her theorem.

It is easy to see that humans have a good understanding of the concept of invariance and symmetry. This is particularly obvious when we consider the rigidity of human-made objects, which is an invariant of Euclidean transformations, or the piecewise rigidity of animal bodies, which can be viewed as a topological invariant. Furthermore, humans have good intuition about the invariance of the laws of Physics. If we try to convince a student that dropping an object onto the floor in one corner of a room will lead to the same outcome as dropping an object onto the floor in the opposite corner of the room, the student probably will wonder whether there is anything unusual in this invariance, simply because this invariance is intuitively so evident that it seems trivial. The student would feel the same about dropping an object onto a floor today and tomorrow.

The invariance of physical laws in the presence of spatial and temporal translations is fundamental, and it has equally crucial implications in the form of conservation laws. Not surprisingly, some examples of the conservation laws are also intuitively obvious, at least qualitatively. Take the intuition that an object dropped from a higher elevation relative to the ground will cause a bigger impact because greater potential energy will translate to greater kinetic energy, consistent with energy conservation laws. Or, when a running person bumps into a standing one, the latter is likely to fall in the direction of the former's movement, which is consistent with conservation of momentum. If, instead of two people colliding, an elastic collision between two billiard balls on a pool table is considered, the higher speed of the first ball will translate to a higher speed of the second ball after the collision, which is consistent with the conservation of kinetic energy.

Humans not only understand invariants and conservations, but they also understand the least-action principles. Everyone knows what it means to complete an action in the shortest amount of time or travel from point *A* to point *B* by using the shortest path. We routinely observe and understand the least-effort or least-energy principle, such as the case of a bobsled run, where the bobsled's trajectory follows the shape of the bobsled slide. The least-action principle has been called a "simplicity" or "likelihood" principle in perception and cognition. Mach (1914) was the first to describe the simplicity and likelihood principles in perception [some consider von Helmholtz (1924/1886), remark in his handbook that familiarity with objects and events disambiguates the visual input as a precursor of Mach's likelihood principle]. The simplicity principle has been a key concept in perception ever since the Gestalt Psychologists adopted and elaborated it (Wertheimer, 1923; Koffka, 1935). The popularity of Gestalt's simplicity principle can be attributed to being intuitively so obvious. When two smooth curves intersect, forming an X, it is nearly impossible to see this intersection as two V's, one upright and the other upside down. An X interpretation is more straightforward!

After listing all these examples of intuitions about invariance, conservations, and least-action, it is surprising how skeptical cognitive psychologists are about intuitive physics (see McCloskey, 1983). Our explanation of this situation is that cognitive psychologists focused on Newtonian physics rather than on Noetherian concepts. Cognitive psychologists have never compared these two probably because our Elementary and High School Physics education has been entirely restricted to Newton's approach. We have the temerity to suggest that the time has come to change both Physics Education and Cognitive Research on Intuitive Physics.

Second, we think that it is essential to reflect that the three concepts from Noether's theorem do generalize outside the context in which the theorem has been formulated. Noether, herself, could not have anticipated this kind of generalization. But this generalization offers, probably for the first time, a possibility of a unified approach to Natural Sciences that include Physics, Cognitive Psychology, and possibly Biology. It should be evident to the reader that Newton's three laws of motion do not offer such a generalization. Specifically, no one ever proposed using Newton's second law, **F** = *m***a**, to explain human visual perception. To be sure, Newton's laws of motion do not contradict Noether's theorem. But the concepts used by Noether in her theorem offer much greater generalizations because they are more abstract. Physical objects are characterized by symmetries. Perceptual and cognitive representations can be treated as conservations. The intermediate stage between the physical and mental worlds, namely the brain's neurophysiological processes, is the stage where the least-action principle is implemented. Poggio et al. (1985) made this suggestion first, and we included references to several papers that explicitly talked about using neural structure to explain perceptual, motor, and cognitive inferences through solving optimization problems. Perceptual inferences are so fast, and they use so little energy, that the physical least-actions are the most likely mechanisms. How exactly is this accomplished? Does the brain have one cost function for all 3D shapes? Do the cost functions change depending on the sensory input? How are the 3D shapes represented in the visual cortex that is mostly a 2D surface? Visual neuroscience has focused for a long time on describing neurons as feature detectors. With our theory, it makes sense to talk about neural structures as circuits solving optimization problems.

Third, are all inferences likely to be veridical, as long as they satisfy our theory? Specifically, let's assume that we identified a symmetry in the physical environment and formulated a cost function that leads to a unique interpretation. Is it always the case that the resulting interpretation will be a conservation rule, in the sense that it will represent something permanent in the environment? It is tempting to think that this might be the case, not only in perception but also in cognition. Can language comprehension be explained that way? Colloquial language is characterized by ambiguities, but humans are extremely good at resolving them. The scientific language is less ambiguous. Can this fact provide clues for how to make the next steps in understanding linguistic communication?

Fourth, it is worth noting that symmetry has a long history in visual arts and esthetics, much longer than the history of symmetry in math and physics. The main difference is that symmetry in art is usually treated as redundancy, whereas symmetry in physics and math is treated as a group invariant. Can visual arts shed additional light on symmetry and contribute to the mathematical formalisms, the way perspective paintings in the fifteenth century stimulated projective geometry in the 17th and 19th centuries?

Fifth, can symmetry be used explicitly in explaining mental representations of objects and concepts? When you look at a chair with a broken leg, you are likely to call it just that, rather than stating that you have never seen such a strange object. Humans tend to form categories around the concept of symmetry and by relation to symmetrical objects. When departures are noticed, they could be explicitly included in the representation as exceptions or treated as noise. This would resemble how the Minimum Description Length principle is used. The mind can use more complex models with less noise to account for a given representation or simpler models with more bits of information devoted to explaining remaining characteristics. This is not a new idea in perception and cognition (see Grünwald et al., 2005; Feldman and Singh, 2006; Grünwald, 2007).

Sixth, the standard topic in vision called “shape from X” can now be treated as part of our theory of “shape from symmetry.” The key step that allows veridical 3D shape recovery from a single 2D retinal image is the redundancy inherent in a mirror-symmetrical object (Pizlo, 2019). But redundancy can be inherent in the sensory data, as well. This is obvious when rigidity constraint has been used by Ullman (1979) in his structure from motion theorem. Rigidity is the invariant of rigid motion, and having two or more views of a rotating object allows for unique shape recovery. Binocular vision is no different. Having two views of the same object is like having two views of a rotating object, except that it is simpler. With a binocular observer, we only have to assume that both eyes look at the same object—rigidity is not invoked. Shape from texture and shading are similar. Gibson's examples of texture gradient are nothing more than translational symmetry of identical texture elements. Without symmetry (redundancy) in the stimulus, there would be no texture gradient and no 3D percept. Multisensory perception integrating vision, audition, and haptics can and should be treated the same way. Different sensors provide redundant (symmetrical) information about the same object or event. By bringing the concept of symmetry to the existing models of “shape from X,” we are not suggesting that computations should be done differently. We think that what we are proposing is similar to Newtonian physics's change to the physics captured by Noether's theorem. The phenomena are the same, but the formalism is more abstract and potentially more powerful.

If our perception approach is accepted, the conventional material in sensation and perception textbooks can be presented differently. The textbooks will not be split into individual visual cues that have to be integrated when a hypothetical binding problem is solved for each symmetrical object and when multiple objects are organized into a scene. Such a perception textbook will be a coherent presentation that can be titled “perception from symmetry,” the same way a modern physics textbook is titled “physics from symmetry” (Schwichtenberg, 2018). Our proposal should not be surprising if we remember that perception is a cognitive capacity to acquire information about the physical world, rather than an analysis of sensory cues isolated from the physical environment.

We conclude by pointing out what might be the most general implication of our approach to perception. Specifically, our emphasis on the relationship between Perception and Physics should encourage vision scientists to think about vision as a 3D phenomenon because the physical environment around us is 3D. The Laws of Physics operate in a 3D real space, not in a 2D camera or retinal image. The Laws of Physics are not invariant in the retinal image; there are no least-action principles nor conservation laws in the retinal image.

## Data Availability Statement

The original contributions presented in the study are included in the article/Supplementary Material, further inquiries can be directed to the corresponding author/s.

## Author Contributions

Both authors listed have made a substantial, direct and intellectual contribution to the work, and approved it for publication.

## Conflict of Interest

The authors declare that the research was conducted in the absence of any commercial or financial relationships that could be construed as a potential conflict of interest.

## Publisher's Note

All claims expressed in this article are solely those of the authors and do not necessarily represent those of their affiliated organizations, or those of the publisher, the editors and the reviewers. Any product that may be evaluated in this article, or claim that may be made by its manufacturer, is not guaranteed or endorsed by the publisher.

## Footnotes

^1^Here we use the standard convention in physics to represent vectors in bold.

^2^*S* is a functional, and not a function, because it depends on the trajectory of the physical system, which is given by a function, say, *x*(*t*). So, *S* is a function of a function, or a functional, that depends on *x*(*t*) and ẋ(*t*), with *x*(*t*) a function of time and ẋ its time derivative.

^3^Technically, Noether's theorem applies to continuous symmetry groups, of which parity is not. But there are arguments that Noether's theorem can also be used for discrete groups. See, e.g., Ashton (2008).

## Supplementary Material

The three appendices showing details of mathematical derivations can be found online at: https://www.frontiersin.org/articles/10.3389/fncom.2021.681162/full#supplementary-material
