# Supplementary material for: The Concept of Symmetry and the Theory of Perception
Source: Front Comput Neurosci. 2021 Aug 23;15:681162. doi: 10.3389/fncom.2021.681162 (PMC8419223; doi:10.3389/fncom.2021.681162)
Supplement: Supplementary file 1 [file Data_Sheet_1.pdf]

Appendix A. Invariance in physics

Newton's laws are invariant under a more general set of transformations called Galilean transformations, including translation as a special case, but also include rotation and uniform motion. To illustrate this, take two observers O and O', and for observer O, a particle's position is represented by the Cartesian coordinates (x, y, z) whereas for O' the same particle's position is represented by (x', y', z'). Assume that, for any particle described by O and O' , the following transformation from (x, y, z) to (x', y', z') holds:

$$\begin{cases} x' &= x + v_{0,x}t, \\ y' &= y + v_{0,y}t, \\ z' &= z + v_{0,z}t, \end{cases} \tag{1}$$

where $v_{0,i}$ are constants. Newton's Second Law relies on the acceleration $\mathbf{a}$ of the particle, which is the same for both observers, i.e., $\mathbf{a} = \mathbf{a}'$, since

$$\ddot{x}' = \frac{d^2 x}{dt^2} + \frac{d^2 \left(v_{0,x}t\right)}{dt^2} = \ddot{x},$$

and similarly for other components. For transformation (1), if Newton's Second Law holds for O, it also holds for O'. However, for another observer O'' whose descriptions

are related to O by, say, the transformation

$$\begin{cases} x'' &= x + v_{0,x}t + \frac{1}{2}a_{0,x}t^2, \\ y'' &= y + v_{0,y}t + \frac{1}{2}a_{0,y}t^2, \\ z'' &= z + v_{0,z}t + \frac{1}{2}a_{0,z}t^2, \end{cases} \tag{2}$$

where $a_{o,i}$ are non-zero constants, Newton's Second Law is not invariant, as

$$\mathbf{a} = \mathbf{a}'' - \mathbf{a}_0 \neq \mathbf{a}''.$$

The reference frames, such as O and O', where Newton's First Law, stating that objects at rest remain at rest if no force acts on them, holds, are called *inertial* reference frames. The set of reference frames, such as O'', where Newton's First Law does not hold, are called *non-inertial* reference frames. For non-inertial reference frames, one needs to add (what Newton called) a fictitious force (in O'' case, $\mathbf{a}_0 m$) to describe the motion of a particle[1]. Thus, Newton's Second Law is invariant under transformations of the type (1) for inertial frames, and not invariant under general transformations, such as that of an accelerated frame.

It is also instructive to add that symmetries of natural laws have physical consequences. Newton assumed that non-inertial reference frames gave rise to fictitious forces, and therefore those were not suitable reference frames. However, Einstein, using his principle of relativity, expanded on the idea that the laws of physics should be invariant for all reference frames, not just inertial reference frames. Einstein was able

[1]Well-known examples of a fictitious force are the centrifugal force and the Coriolis force.

to generalize the range of application of dynamical equations by imposing a larger group of symmetry to physical laws, thus creating his general theory of relativity. What to Newton was a fictitious force was, for Einstein, the actual manifestation of space-time curvature.

We should point out that invariance does not mean that the equations describing a physical system are invariant themselves. For example, the laws of motion are the same for two observers in uniform motion with respect to each other: they apply to an observer O and an observer O' moving with respect to O with a constant velocity $v_{O'}$ in the x-direction. But the description under O and O' of the same object can be different. For example, an object at rest with respect to O will be described in O' as having non-zero velocity $\mathbf{v}' = -v\hat{\mathbf{x}}$. The equations of motion are not invariant, but they transform with the coordinate systems of O and O' and are thus called covariant.

Appendix B. Derivation of EL equation

Here we show in more detail how the Euler-Lagrange equations can be derived from the variational principle. As discussed in Section 3, we start with the action integral and make small perturbations $\delta q_i(t)$ to the actual trajectory of the particle, $q_i(t)$.

The perturbations $\delta q_i\left(t\right)$ induce a perturbation in the value of the functional S given by[2]

$$\begin{aligned} \delta S &= \delta\left[\int_{t_1}^{t_2} L\left(q_i, \dot{q}_i, t\right) dt\right] \\ &= \int_{t_1}^{t_2} \delta L\left(q_i, \dot{q}_i, t\right) dt \qquad (3) \\ &= \int_{t_1}^{t_2} \left[L\left(q_i + \delta q_i, \dot{q}_i + \delta\dot{q}_i, t\right) - L\left(q_i, \dot{q}_i, t\right)\right] dt, \qquad (4) \end{aligned}$$

where

$$\delta\dot{q}_i = \delta\frac{dq_i}{dt} = \frac{d\delta q_i}{dt}.$$

Assuming that the perturbations δq_i are very small, i.e. that we are keeping the perturbed trajectory close to the actual q_i, we can expand (4) in terms of the

[2]For simplicity, we are dropping the explicit dependency of q_i and $\dot{q}_i$ on t from our notation, but the reader should keep in mind that both quantities are a function of t.

perturbations δq_i and $\delta \dot{q}_i$, namely

$$\begin{aligned}
\delta S &\approx \int_{t_1}^{t_2} \left\{ \left[L + \sum_i \frac{\partial L}{\partial q_i} \delta q_i + \sum_i \frac{\partial L}{\partial \dot{q}_i} \delta \dot{q}_i \right] - L \right\} dt \\
&= \int_{t_1}^{t_2} \left[\sum_i \frac{\partial L}{\partial q_i} \delta q_i + \sum_i \frac{\partial L}{\partial \dot{q}_i} \delta \dot{q}_i \right] dt \\
&= \int_{t_1}^{t_2} \left[\sum_i \frac{\partial L}{\partial q_i} \delta q_i + \sum_i \frac{\partial L}{\partial \dot{q}_i} \frac{d\delta q_i}{dt} \right] dt. \qquad (5)
\end{aligned}$$

If we integrate by parts the second summation term of (5), we get

$$\begin{aligned}
\int_{t_1}^{t_2} \sum_i \frac{\partial L}{\partial \dot{q}_i} \frac{d\delta q_i}{dt} dt &= \int_{t_1}^{t_2} \left[\sum_i \frac{d}{dt} \left(\frac{\partial L}{\partial \dot{q}_i} \delta q_i \right) - \sum_i \frac{d}{dt} \left(\frac{\partial L}{\partial \dot{q}_i} \right) \delta q_i \right] dt \\
&= \sum_i \left(\frac{\partial L}{\partial \dot{q}_i} \delta q_i \right) \Bigg|_{t_1}^{t_2} - \int_{t_1}^{t_2} \sum_i \frac{d}{dt} \left(\frac{\partial L}{\partial \dot{q}_i} \right) \delta q_i dt \\
&= - \int_{t_1}^{t_2} \sum_i \frac{d}{dt} \left(\frac{\partial L}{\partial \dot{q}_i} \right) \delta q_i dt,
\end{aligned}$$

where in the last line we used the assumption that $\delta q_i \left(t_1\right) = \delta q_i \left(t_2\right) = 0$. Substituting the above result in (5) we obtain

$$\begin{aligned}
\delta S &\approx \int_{t_1}^{t_2} \left[\sum_i \frac{\partial L}{\partial q_i} \delta q_i - \sum_i \frac{d}{dt} \left(\frac{\partial L}{\partial \dot{q}_i} \right) \delta q_i \right] dt \\
&= \sum_i \int_{t_1}^{t_2} \left[\frac{\partial L}{\partial q_i} - \frac{d}{dt} \left(\frac{\partial L}{\partial \dot{q}_i} \right) \right] \delta q_i dt. \qquad (6)
\end{aligned}$$

It follows from (6) that, since δq_i are arbitrary and independent perturbations, in order for S to be minimized, i.e. $\delta S = 0$, the following set of equations need to be satisfied:

$$\frac{\partial L}{\partial q_i} - \frac{d}{dt}\left(\frac{\partial L}{\partial \dot{q}_i}\right) = 0. \tag{7}$$

Eqs. (7) are known as Euler-Lagrange (EL) equations, and their solution minimizes the action S between arbitrary times t_1 and t_2.

Appendix C. Conservation of energy

In this Appendix we show how, for a simple example, conservation of energy can be obtained from the invariance of the Lagrangian under time translations.

Let us return to the classical particle constrained to move in the direction x. Let K and V represent the kinetic and potential energies of this particle, with $K = \frac{1}{2}mv_x^2$ and $V = V(x)$ a function of the particle's position. The action that will be minimized is defined as follows:

$$S = \int_{t_1}^{t_2} (K - V)\, dt. \tag{8}$$

As before, the integral in equation (8) is to be computed through the trajectory of the particle between two points, $x(t_1)$ and $x(t_2)$, but, to simplify the argument, we will follow Hanc, Tuleja, and Hancova (2004) explanation. Hanc et al. computed S between times t_1 and t_3 by breaking it into two segments: $A = (t_1, t_2)$ and $B = (t_2, t_3)$, with $t_1 < t_2 < t_3$, $(t_2 - t_1) \ll 1$, and $(t_3 - t_2) \ll 1$. In other words, we are computing (8) for two infinitesimal segments A and B, and because of our construction, we can use a discrete approximation. The idea here is to break down a complicated variational problem into a simpler approximation with only two infinitesimal segments, such that we can see the idea behind it.

Let m be the particle's mass, and let us assume that the particle moves under an interaction represented by the potential $V(x)$. When we compute the kinetic energy K, we may approximate the speed $v_x = \dot{x}$, for small time intervals, as $v_x \approx (x_2 - x_1) / (t_2 - t_1)$. For the time interval (t_1, t_2), the potential energy V should be evaluated at the average position of the segment (x_1, x_2). Thus, the integral in (8)

can be approximated, for the small time interval (t_1, t_2), as

$$S_A = \frac{1}{2} m \frac{(x_2 - x_1)^2}{(t_2 - t_1)^2} (t_2 - t_1) - V \left(\frac{x_2 + x_1}{2} \right) (t_2 - t_1) \tag{9}$$

Similarly, for the next segment B:

$$S_B = \frac{1}{2} m \frac{(x_3 - x_2)^2}{(t_3 - t_2)^2} (t_3 - t_2) - V \left(\frac{x_3 + x_2}{2} \right) (t_3 - t_2) \tag{10}$$

The action for the interval (t_1, t_3) is $S = S_A + S_B$. First note that, since V is time-independent, the action S is invariant under time translations, since the time integral of the Lagrangian depends on time differences, and not on the absolute time. In other words, absolute time is not present either in the Lagrangian $(K - V)$ or in the action S. This is similar to our example above, where we derived the conservation of momentum directly from the EL equation by assuming that the Lagrangian does not depend on the position x. Thus, adding a constant to all times will not change S. So, S is symmetric under time translation. Now comes the essential part, where we evaluate, following Noether's theorem, which conservation law is related to time symmetry. Once again, following the derivation in Hanc et al. (2004), we evaluate the first derivative of S with respect to t_2 in the intermediate point x_2, and we set this derivative to zero, which is the necessary condition for the minimum of S,

$$\left. \frac{dS}{dt} \right|_{t=t_2} = \left. \frac{d (S_A + S_B)}{dt} \right|_{t=t_2} = 0. \tag{11}$$

If we substitute (9) and (10) in (11) and differentiate with respect to t in t_2, we obtain

$$\frac{1}{2}m\frac{(x_2 - x_1)^2}{(t_2 - t_1)^2} + V\left(\frac{x_1 + x_2}{2}\right) = \frac{1}{2}m\frac{(x_3 - x_2)^2}{(t_3 - t_2)^2} + V\left(\frac{x_2 + x_3}{2}\right). \qquad (12)$$

Equation (12) has a clear interpretation: it shows that the total sum of the potential and kinetic energy in segment A is the same as in segment B. So, equation (12) expresses the conservation of mechanical energy. This is the gist of how conservation of energy is derived from symmetry under time translation. Here, time and total energy are conjugate variables.

References

Hanc, J., Tuleja, S., & Hancova, M. (2004). Mental rotation of three-dimensional objects. *American journal of physics*, *171*(3972), 428-435.
